# The Multiple Object Avoidance (MOA) task measures attention for action: Evidence from driving and sport

**DOI:** 10.3758/s13428-021-01679-2

**Published:** 2021-11-16

**Authors:** Andrew K. Mackenzie, Mike L. Vernon, Paul R. Cox, David Crundall, Rosie C. Daly, Duncan Guest, Alexander Muhl-Richardson, Christina J. Howard

**Affiliations:** 1 Department of Psychology, Nottingham Trent University, Nottingham, NG1 4FQ UK; 2 School of Design and Informatics, Abertay University, Dundee, DD1 1HG UK; 3 Department of Psychology, Cambridge University, Cambridge, CB2 3EB UK

**Keywords:** Visual cognition, Driving, Visual attention, Multiple object tracking, Multiple object avoidance, Sport

## Abstract

Performance in everyday tasks, such as driving and sport, requires allocation of attention to task-relevant information and the ability to inhibit task-irrelevant information. Yet there are individual differences in this attentional function ability. This research investigates a novel task for measuring attention for action, called the Multiple Object Avoidance task (MOA), in its relation to the everyday tasks of driving and sport. The aim in Study 1 was to explore the efficacy of the MOA task to predict simulated driving behaviour and hazard perception. Whilst also investigating its test–retest reliability and how it correlates to self-report driving measures. We found that superior performance in the MOA task predicted simulated driving performance in complex environments and was superior at predicting performance compared to the Useful Field of View task. We found a moderate test–retest reliability and a correlation between the *attentional lapses* subscale of the Driving Behaviour Questionnaire. Study 2 investigated the discriminative power of the MOA in sport by exploring performance differences in those that do and do not play sports. We also investigated if the MOA shared attentional elements with other measures of visual attention commonly attributed to sporting expertise: Multiple Object Tracking (MOT) and cognitive processing speed. We found that those that played sports exhibited superior MOA performance and found a positive relationship between MOA performance and Multiple Object Tracking performance and cognitive processing speed. Collectively, this research highlights the utility of the MOA when investigating visual attention in everyday contexts.

## General introduction

Performance in everyday tasks, such as driving and sport, requires appropriate allocation of attention to task-relevant information and the ability to inhibit task-irrelevant information. Yet the ability of this attentional control varies across individuals. Where, for example, there are differences in the speed of attentional processing, the number of objects one can attend to or the ability to successfully inhibit attentional information. There are countless tasks designed to target these and other attentional components, often with the aim to assess an individual’s attentional control and how this relates to performance in more complex tasks. The overall aim of this research was to investigate a novel, open-source, visual-attention task called the Multiple Object Avoidance (MOA) task to assess visual attention function and demonstrate its relatedness to attention in everyday tasks. This is a visuomotor task that was developed with the aim of creating a more active (i.e. involving visuomotor control) Multiple Object Tracking (MOT) task – a task that is often used in attention research given the proposed attentional similarities to complex everyday tasks. The MOA task was originally developed in response to previous research in driving. Mackenzie and Harris (2017) found that MOA performance positively predicted driving performance and eye-movement scanning in a driving task. The findings in that study highlighted the potential importance of such a task in predicting driving behaviour but had several limitations including the absence of an open-source version of the task. As such, this research is presented that continues the line of MOA and driving literature before also exploring the utility of MOA in further everyday domains; that of sport.

We first discuss the importance and development of the MOA in relation to “active vision” in the next section. In Study 1, we discuss the literature on visual attention and driving behaviour with a specific focus towards a more “action-related” visual assessment of these aspects. In this study, we aimed to explore the efficacy of the MOA task in predicting driving performance and hazard perception when driving. In Study 2, we explored the MOA in a sporting domain and aimed to investigate MOA performance differences in those that play sports – a population that has often been found to exhibit superior visual attentional function – and those that do not play sports. In order to establish a degree of construct validity we also aimed to investigate the attentional relatedness of the MOA to other cognitive tasks argued to be important in sporting performance.

### A role for active visual attention tasks and the development of the MOA

Given the attentional complexity of “everyday tasks” (such as driving and sport), it is unlikely a task measuring a single attentional domain would predict overall task performance (Bowers et al., 2013; Liebherr et al., 2019). Often, the relatedness, or lack thereof, of the cognitive task or battery to the attentional demands of the everyday task limits its efficacy in predicting task performance. In the case of driving and sport, whilst elements of, for example, selective attention or executive control are important and could be assessed using tasks such as Stroop tasks or inhibitory response tasks, one must also have the ability to sustain and divide attention to dynamic stimuli (Alberti et al., 2014; Bowers et al., 2011; Mackenzie & Harris, 2017) which these types of selective attention tasks do not capture.

One task that may capture the range of attentional complexity in sport and driving is the Multiple Object Tracking task (MOT) (e.g. Cavanagh & Alvarez, 2005; Pylyshyn & Storm, 1988). In a simple MOT task, target and distractor objects are presented on-screen. Observers are asked to attend to all the targets. The target objects are usually denoted as such by a temporary increase in visual salience of the object e.g. by flashing or changing colour. Observers must continue to divide their attention across all target objects as they and the distractors move around the visual scene. Once the objects have stopped, observers must identify which of the objects were originally the targets. One might hypothesise that performance on this task correlates to complex everyday behaviour; in tasks that involve sustained and divided attention to multiple dynamic stimuli whilst ignoring distractor stimuli. Indeed, this has been found in a number of studies where poorer performance in an MOT task correlated to poorer performance on road tests, and poorer ability to detect pedestrians during simulated driving (Alberti et al., 2014; Bowers et al., 2011). Importantly, in the work of Alberti et al., (2014), MOT was a stronger predictor of pedestrian detection than the Useful Field of View (UFOV) task, which is a more reduced task that does not capture the sustained and dynamic elements of attention in everyday tasks. Michaels et al. (2017) investigated the relationship between individuals’ perceptual-cognitive capacity in a MOT task and driving behaviour. They found that individuals who performed more poorly in the MOT task were at a higher crash risk – particularly in older adults. Collectively, these results highlight the link between visual attentional function and task performance, and also the possible importance of using a more dynamic and sustained attention assessment in predicting behaviour.

Mackenzie and Harris (2017) argued that whilst the MOT likely captures attentional properties involved in driving more than tests such as the UFOV, it is still relatively passive in nature because there is no active, visuomotor interaction during the motion phase. Indeed, one of the better strategies to use in order to be successful in the MOT task is to make fewer eye movements and covertly attend to the stimuli by attempting to fixate centrally between the moving targets (Fehd & Seiffert, 2008; Oksama & Hyönä, 2016; Zelinsky & Neider, 2008), so that even the active exploration with the eyes is reduced. In driving, for example, there is a visuomotor element in controlling the vehicle (Kountouriotis et al., 2012; Land & Lee, 1994; Lehtonen et al., 2014) and one must make many eye movements to successfully identify hazards (Konstantopoulos et al., 2012; Underwood et al., 2002, 2005). Eye movements, attention, and action are often intrinsically linked (Hommel, 2010; Humphreys et al., 2010) particularly in everyday settings, including driving (Land, 2006; Tatler et al., 2011) and sport (Land & McLeod, 2000). In addition, different eye-movement strategies are observed between tasks involving action (visuomotor control) and their passive analogies, e.g. ‘real life’ versus video (Foulsham et al., 2011; Mackenzie & Harris, 2015; Risko et al., 2012). Thus, we argue, visual attention tasks incorporating the more active elements of attention may better predict performance.

Attempts have previously been made at capturing this more action-related element of visual attention by developing an interactive MOT task or iMOT (Thornton et al., 2014; Thornton & Horowitz, 2015). In this task, the individual must use a touch screen to move objects and prevent them colliding with each other. Following from this research, Mackenzie and Harris (2017) identified a task similar in nature that did not involve a touch screen element and only involved the control of one object (similar to driving) using a mouse. A non-touch screen design prevented obstruction of the screen from hands and arms. They termed this task the Multiple Object Avoidance (MOA) task. In this task, an individual controls one object (user-controlled object). Three other objects (red hazard balls) are present on screen and begin moving around. The task is to have the blue object avoid these red hazard objects that would move in a predictable, vector-like fashion. As the individual continues to manoeuvre the user-controlled object to avoid the hazard objects, the task gets increasingly harder as more red balls are added (one added every 10 s).

Arguably, a task like this involves similar attentional components to those used in complex everyday tasks. Namely, sustained attention to dynamic stimuli, divided attention, active vision, visuomotor control, and planning ability (i.e. the ability to predict the motion of the objects). In Mackenzie and Harris' (2017) work, performance on this MOA task significantly predicted driving performance and also predicted more effective horizontal spread of visual search – eye movement behaviour we typically see in more experienced drivers (Crundall & Underwood, 1998; Konstantopoulos et al., 2010; Konstantopoulos et al., 2012; Underwood et al., 2011). This relationship was stronger for MOA than a standard MOT task and was also stronger during more complex scenes; scenes that would intuitively involve more scanning type eye movements to detect hazards. They explain this relationship by suggesting that the active nature of the MOA which involves many eye movements to be successful may represent the eye movements one makes when driving and searching for hazards. This is important given that inattention and failures to scan the road are often contributing factors to accidents (Dingus et al., 2006; Lee, 2008).

The broad aims of this research are to replicate and extend Mackenzie and Harris (2017) and investigate how MOA predicts driving and driving related behaviours using a newly developed open-source version of the MOA task (Study 1) and begin exploring how MOA performance might differentiate between those with varying sporting expertise (Study 2).

## Study 1: The Multiple Object Avoidance task and driving behaviour

Driving is a complex visuomotor everyday task. It requires the ability to control the vehicle whilst also attending to possible hazards. One’s own visual attentional functioning (that is, performance within specific facets of visual attention e.g. divided attention, speed of processing, working memory capacity etc.), is often therefore a predictor of driving behaviour and driving performance. For example, better ability within these visual attentional components relates to better driving overall and the individual elements of driving, e.g. hazard perception (Wood et al., 2016), vehicle control (Aksan et al., 2017; Louie & Mouloua, 2019) and, importantly, road accidents (Karimi et al., 2015). Thus, the importance of investigating and evaluating visual attention tasks that may help to predict, assess, or even train, driving behaviour is highlighted. In this study, we aimed to replicate and extend the results of Mackenzie and Harris (2017) by exploring the MOA task’s ability to predict simulated driving performance and hazard perception behaviour, and also how it may relate to other measures used in driving such as the Useful Field of View and the Driving Behaviour Questionnaire.

### Measuring visual attentional functioning and driving performance

The relationship between visual attentional function and driving ability is evident in a number of studies where superior driving performance is predicted by superior performance in tasks measuring, for example, overall executive functions (Pope et al., 2016), processing speed (Ross et al., 2016) and sustained attention (Tabibi et al., 2015). Early work demonstrates how the Useful Field of View (UFOV) test (Ball et al., 1990) relates to driving behaviour and performance – particularly in older adults. Broadly, this test involves a range of executive functions measuring the ability to process multiple (divided attention) rapidly presented pieces of information (speed of visual processing) whilst ignoring distractors (executive control). Better performance in this test seems correlated to better driving performance, at least in older adults (Ball et al., 1993; Bedard et al., 2008; Clay et al., 2005; Owsley & McGwin Jr., 2010). The link between visual attention function and driving found with many UFOV studies (and other attention task studies) can perhaps be explained by the attentional similarities in what is required in driving and the UFOV task. In driving, one must also be able to process visual information effectively (e.g. hazards), divide attention to several elements of the environment (e.g. control of the vehicle, looking out for hazards etc) and, importantly, ignore distractors. One may argue that if a driver exhibits better attentional function, then they are better able to handle these attentional demands of the road.

It is, however, important to also highlight that some of the relationship between visual-cognitive tools and driving performance in older adults may simply reflect normal ageing. Bédard et al. (2016) investigated this idea using the ANT (Attentional Network Task) and UFOV tasks by running correlations between age and task performance within certain age groups (under 65 and over 65) rather than using the full age range of participants. When age was partialled out, correlations in task performance within these age groups disappeared. In the case of UFOV we know that visual processing speed (which the UFOV largely measures) is a cognitive function where the variability in processing speed is lower within younger populations, the decline is measurably marked with age and there are large differences between younger and older populations (Guest et al., 2015, 2017). It is therefore unsurprising that such a task would capture attentional and driving differences when used across these age groups. The research by Bédard et al. (2016) demonstrates that a large amount of the variability in task performance is simply accounted for by age. It also highlights issues in developing cognitive tasks to predict driving behaviour. We attempt to address this limitation here by developing a tool that correlates with driving performance within a younger adult population where variability of cognitive decline due to normal aging is unlikely to contribute to the variability in task performance.

Nevertheless, there has been some evidence that links performance in paradigms utilising static or brief presentation of stimuli to driving behaviour within adult (non-older) populations. Paradigms such as, for example, the Deceleration Detection Flicker Task, a task that measures ability to respond to a perceived reduction in driver headway (see Crundall, 2009; Lee et al., 2020), and the Attentional Network Task (ANT), a task that measures attention alerting, attention orienting and executive control (Fan et al. 2002, 2009). Weaver et al. (2009) conducted a study comparing performance on the ANT to both simulated and on-road driving performance. They found moderate relationships between overall ANT performance and driving scores (although this was stronger for simulated driving). However, the strength of the relationships between the individual attentional components and driving scores were quite weak. This may be surprising given that these attentional components would likely be used in driving where one must, for example, be vigilant for oncoming hazards (attention alerting), orient attention to potentially hazardous areas (attention orienting) and attend to hazardous areas whilst ignoring non-hazardous areas of the scene (executive control). However, in the study, they used a more general measure of driving (i.e. starting, stopping, signal violations, right of way violations) and a non-hazardous driving route that may not have been sensitive to measure performance in specific driving tasks where these attentional function components are arguably more vital (e.g. hazard perception). Roca et al. (2013) therefore investigated how performance in a version of the ANT predicted attention to specific hazardous events (hazards predicted by a single precursor). They found that attention orienting specifically was the best predictor of safe driving behaviour during specific hazardous events. Therefore, one might argue that tasks such as the ANT might do well in predicting behaviour during more specific and low occurrence road events (e.g. hazards) but may not capture the attentional complexity in more general driving. Our aim is to therefore extend beyond identifying how a task might predict specific driving events and develop a task that might better predict general driving performance. We believe, given the MOA’s targeting of active and divided attention to dynamic stimuli, it is a suitable candidate.

#### Aims and hypotheses

Mackenzie and Harris (2017) attempted to address the limitations of paradigms utilising static or brief presentation of stimuli as assessments of visual attention in relation to driving. Namely, limitations surrounding these tasks’ inability to capture general driving behaviour and surrounding the minimal contribution of more sustained and dynamic elements of visual attention in such tasks. They proposed the MOA task (Section 1.1.1) and found performance in this task did well in predicting driving performance in complex (e.g. urban) driving environments and also visual scanning, where better performance in MOA predicted better driving performance and wider horizontal scanning. However, there were a number of limitations identified and these are addressed in this first study. Importantly, we address these limitations using a new open-source version of the MOA task.

The first aim (1a) was to replicate the previous work of Mackenzie and Harris (2017) investigating how well performance in the MOA predicts simulated driving performance and to extend this by making a comparison with the predictive power of the UFOV; a more frequently used measure of attention in the driving literature. This was done by measuring performance on the MOA and UFOV and correlating scores, via regression, with driving performance in a driving simulator. We hypothesised that better MOA performance would predict better simulated driving performance.

The second aim (1b) was to identify how well performance in this task predicts hazard perception behaviour. Mackenzie and Harris (2017) previously claimed that being able to predict horizontal spread of visual search was an advantage in the MOA as this eye movement behaviour *may* help to identify hazards. However, there were no hazards present in that study to provide evidence for this. Therefore, we investigated if performance in the MOA (and UFOV) predicted the time to first fixate hazards during the simulated drive. We hypothesised that better MOA performance would predict earlier hazard first fixation times and this relationship would be stronger for the MOA than the UFOV because of the increased oculomotor activity required during the MOA.

The third aim (1c) was to investigate some measures of reliability and validity in the MOA. We did this by investigating the test–retest reliability (after 6 months) and investigated the concordant validity of this test with typically used self-report measures in driving using the Driving Behaviour Questionnaire (DBQ). We hypothesised there to be modest test–retest reliability. We also hypothesised there to be moderate correlations between the MOA and scores on the DQB where higher MOA scores would correlate to fewer instances of self-reported driving errors for each subscale of the DBQ.

## Method

### Participants

Forty-two participants (11 males; 31 females) with a mean age of 23.26 years (SD = 4.35) took part in this study. All participants held a valid driver’s licence (M = 4.44 years; SD = 4.05), drove on the left (e.g. United Kingdom) and had normal or corrected-to-normal vision (via contact lens). Participants were paid in £20 shopping vouchers for their participation. A sample calculation was conducted in *R* using the package *pwr (v.1.3-0)*, which contains functions for basic power calculations using effect sizes and notations from Cohen (1988). A predicted effect size of Cohen *f*^2^ = 0.4 was used using Mackenzie and Harris’ (2017) previous data where significant effect sizes ranged from *f*^2^ = 0.20 and *f*^2^ = 0.59. At a more conservative power level of 0.95, alpha error probability of 0.05 and three modelled predictors (theoretically; two predictors and one covariate), a sample of 45 participants would be recruited. For this, we note the limitation of being underpowered here in obtaining this effect size. For a power level of 0.8, alpha error probability of 0.05 and three modelled predictors, a sample of 29 participants would be recruited. For the MOA retesting, 28 of the participants were successfully recruited (nine males). There was no significant difference between the ages of participants between the original 42 participants (M = 23.26, SE = 0.67) and the 28 participants who returned for retesting MOA ((M = 24.39, SE = 0.87), *t*(56.14) = – 1.04, *p* = 0.30). Additionally, the difference in driving experience (years) between the original 42 participants (M = 4.44, SE = 0.63) and the 28 participants who returned retesting MOA (M = 5.41, SE = 0.86) was not significant (*t*(53.54) = – 0.92, *p* = 0.36). Ethical approval was given by Nottingham Trent University College Research Ethics Committee.

### Stimuli and apparatus

#### Visual attention tasks

##### Multiple Object Avoidance (MOA) task

**Fig. 1:**
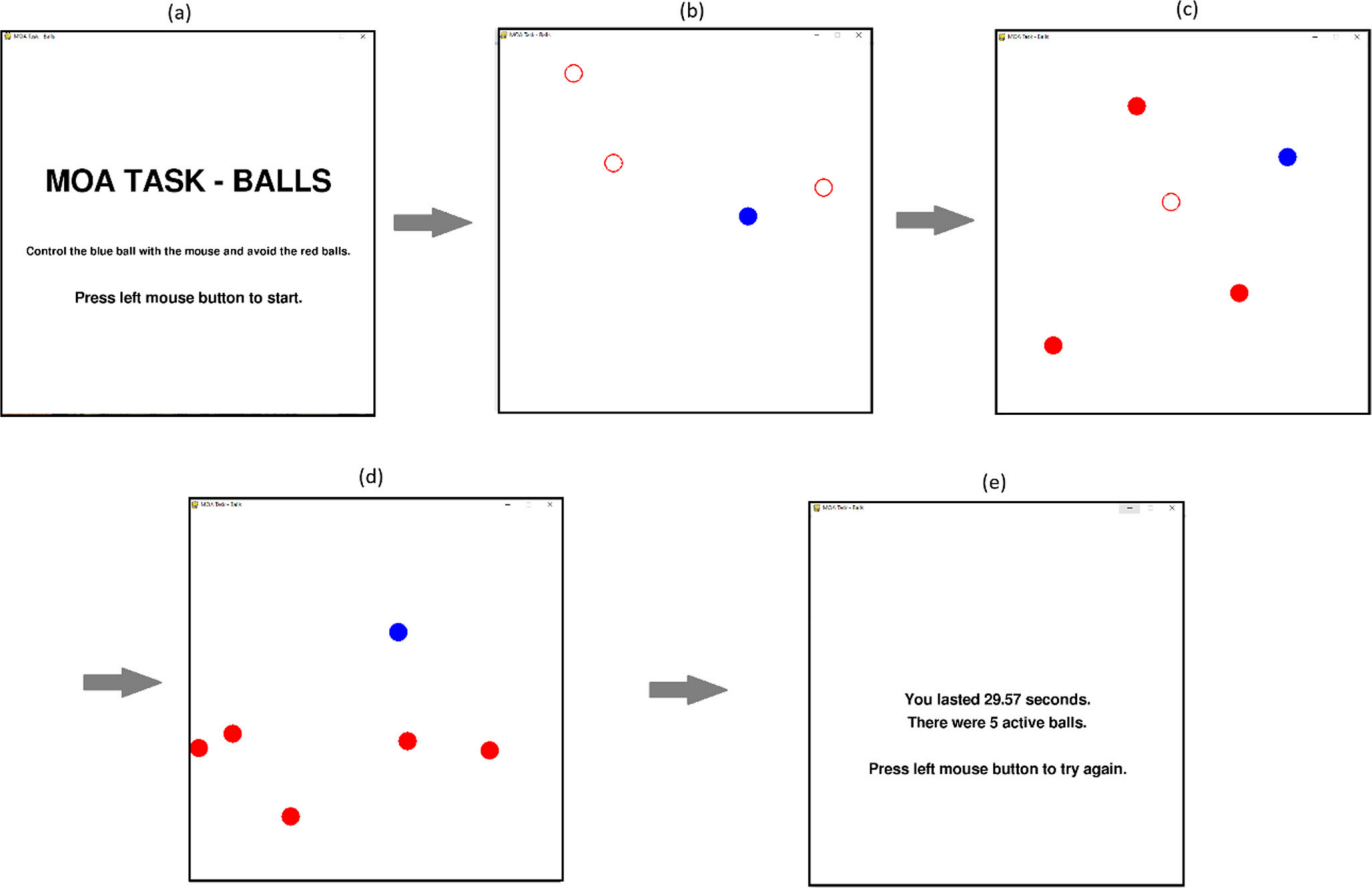
The MOA task. Participants are presented with a start screen and must press the mouse button to continue (**a**). Initially, the red hazard circles will appear unfilled for one second to allow participants the time to identify their speed and trajectory (**b**). The trial will not end if the blue circle collides with the red circles in this state. After 10 s, a new red hazard circle is added (**c**). It remains unfilled for 1 s. This sequence is repeated (**d**) until the participant’s blue circle collides with one of the solid red circles. A feedback screen is presented, and the experiment notes the time of the trial (**e**)

This task was programmed using Python and the packages: pygame, numarray, numeric, and numpy. Initially, four circles are presented on screen, each 40 pixels in diameter (~10.6 mm) size. One of these is blue and three are red. The blue circle is controlled by the participant using a mouse and the objective is to avoid the red hazard circles touching the blue circle as they move around the screen. There is an initial delay of 1 s where the red circles begin to move but are unfilled and unable to collide with the user’s blue circle. This is to give the participants time to identify the stimuli and their trajectory. The hazard circles are then filled in and, at this point, can collide with the blue user-controlled circle. After 10 seconds another moving red circle is added to the display. It initially appears as an unfilled circle for 1 s and is unable to collide with the user’s blue circle before being filled in red completely. All red circle movements followed predictable straight-line vector movements after an initial random trajectory. That is, a red circle will move in a straight line until it connects with either the edge of the screen or another red ball and ‘bounce’ off this object at an angle consistent with 2D vector physics. No red object moves in a random pattern after the initial trajectory and, as such, all movements are theoretically predictable. Speeds for each red circle are randomised and can range from any number from 0 to 680 pixels per second. A new red circle is added every 10 s, thereby increasing the difficulty of the task with more objects to track and avoid (see Fig. 1 for a sequential representation). The time (in seconds) that a participant can avoid colliding with a red circle is recorded as the score for the trial, with higher numbers reflecting greater proficiency at the task. In this study, participants completed ten trials; two practice trials and eight recorded trials. A mean of these final eight trials is taken as the measure of MOA performance. The task window is displayed at a size of 800 by 800 pixels. The task was presented on a 17.5-inch CTX EX951F monitor (Chuntex Electronic Co., Ltd., Taipei, Taiwan) with a refresh rate of 85 Hz.

##### Useful Field of View (UFOV)

**Fig. 2:**
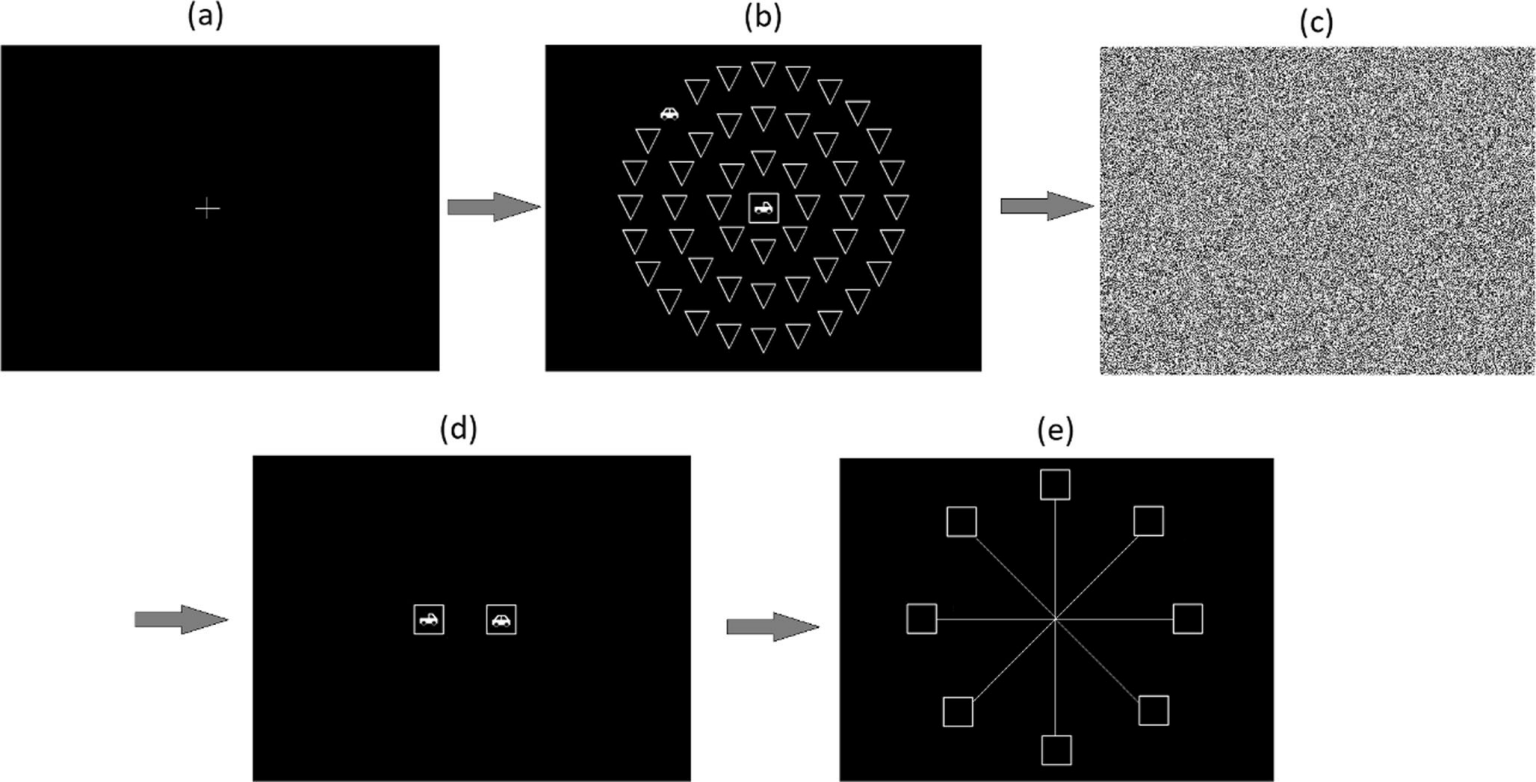
Sequential view of UFOV subtask 3. Participants fixate centrally (**a**) before being presented with the image in (**b**) at varying durations. After this image, participants must select, using a mouse, which object appeared centrally, either a car or truck (**d**), and then where the peripheral object appeared (**e**)

Version 7 of the Useful Field of View task was used (Brain HQ, Posit Science). There are three subtasks. Subtask 1 one measures speed of processing to a single object. An image of either a car or truck appears in the centre of the display screen and it is the participant’s task to identify which object is presented. The duration for which the stimuli is presented at varies, in a stepped fashion, depending on the accuracy of identification (where better performance per trial results in shorter presentation durations). Subtask 2 measures divided attention where the central task remains the same as in Subtask 1, but the participant must identify where another target is on screen. Subtask 3, which measures divided attention amongst distractors, is the same as Subtask 2 but a number of distractors (triangle shaped stimuli) appear in the field of view (Fig. 2). Processing speed, as measured by the software depending on stimuli presentation duration, is used as a measure of performance in each subtask. Of interest for this study are the scores from subtask 3. Arguably Subtasks 1 and 2 are more relevant for Older Adult drivers and a ceiling performance was observed here in our sample for these subtasks. Subtask 3 – divided attention amongst distractors – is more relevant to attention in driving in a younger adult population and will show variance across younger participants. Note: presentation durations and processing speed calculations were all controlled by the UFOV software. Experimenters did not have access to the raw data for these and scores are not given for each trial individually. Performance is a measure of processing speed and is measured as the minimum amount of time required to correctly process the visual information where better performance results in lower minimum speed (in ms). The task was presented on a 17.5-inch CTX EX951F monitor (Chuntex Electronic Co., Ltd., Taipei, Taiwan) with a refresh rate of 85 Hz and at a screen resolution of 1280 x 1024.

#### Driving simulation

**Fig. 3:**
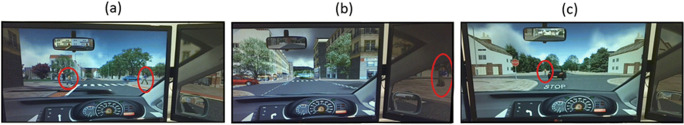
Examples of driving environment and the three hazardous situations. For each, the pedestrian steps onto the road without looking or hesitating and would require the driver to either slow down or stop to avoid a collision

A RijSchoolSimulator driving simulator, developed by Carnetsoft, was used for the driving simulation aspect of the study, as used in previous studies on driving behaviour (Roca et al., 2018; Tejero et al., 2019). The hardware includes a Logitech G27 control set featuring an 11-inch leather wrapped driving wheel; 6-speed gear shifter (including reverse); steel accelerator, brake, and clutch pedals (Fig. 3a). Three display monitors provide a 210-degree horizontal field of view from the cabin in frontal and side view positions. Mirrors, dashboard, and road environment were all displayed across a three-screen panel display (Fig. 3b). The software allows for the preparation and testing of behavioural experiments. The graphic capabilities of the simulator are able to portray a 3D world, rear-view and side-view mirrors, as well as visual and sound effects to simulate changing weather conditions (see Fig. 3 for a representation of the visual field).

The software allows for interactive traffic in the form of moving vehicles as well as animated pedestrians and animals. Participants completed three driving routes of varying complexity. The urban route consisted of typical inner-city driving involving crossroads, single-lane traffic, traffic lights, pedestrians etc. This was the most complex route. The next complex route was a suburban carriageway. This consisted of both single and dual lane roads, junctions and a number of speed limit changes. The final, and least complex route was the inter-city motorway. This consisted of a multi-lane carriageway in a straight line. There was a moderate level of traffic throughout. Driving performance was tracked by the driving simulator throughout. This was a point-deduction system (as opposed to a demerit-based point system that has been previously used e.g. Mackenzie & Harris, 2017; Weaver et al., 2009) where points were deducted, starting from a score of 10, for driving error. Driving assessment included elements such as speed control, lane changing, rules of priority, gear changing, overtaking, steering, indicator usage, and negotiating roundabouts. All scores were controlled by the software. Each of the individual assessment tasks are rated as on a decimal scale from 0 to 10. Scores were recorded for each route and for all three combined (as an average of the three routes). Three hazards were programmed in the urban route. These were pedestrian-based hazards where a pedestrian would step into the road (Fig. 3).

### Eye movement recording

Eye movements were recorded using SMI eye tracking glasses (ETG2), sampling binocularly at 60 Hz. The environment is captured using a forward-facing camera sampling at 60 fps. A standard one-point calibration was used using a circular target presented on-screen before each drive. Participants were free to move their head naturally as they drove. Eye movements were automatically overlaid onto forward-facing video by the eye tracking hardware.

The times to first fixate the hazards and hazard precursor were taken as our measures of hazard perception. The precursor for the three hazards are behavioural in nature (Crundall et al., 2012) whereby the precursor is the same stimulus as the hazard and behaves in a manner that allows for future projection of the hazard nature. In this instance, the pedestrians walk towards the road without slowing down, stopping or looking at approaching traffic. The time taken to fixate on precursors has been shown to discriminate between experienced and inexperienced drivers (Crundall et al., 2012). The precursor period is defined as the time between when the pedestrians enter the image and the frame before they step onto the road. Hazard onset times began on the frame pedestrians stepped into the road.

Eye movements were manually coded using “semantic gaze mapping”. This is a method used in real-time eye movement capture to attribute eye movements to a semantically meaningful area of interest. In this instance, the semantic categories of ‘hazard’ and ‘precursor’ were identified. The experimenter manually identified which fixations landed on each of the hazards and precursors and would, using the SMI BeGaze software, assign these to the area of interest. First fixation times for hazards were calculated as the time to first fixate on the hazard minus the hazard onset time. First fixation times for precursors were calculated as the time to first fixate on the precursor minus the time in which the precursor is first available in the field of view.

### Questionnaire measures

The Driving Behaviour Questionnaire (DBQ) was used to measure self-report driving behaviour (Reason et al., 1990). We used the 28-item questionnaire with four subscales: Aggressive Violations, Ordinary violations, Attentional lapses and Errors. Each item describes a particular driving behaviour and participants rate on a Likert scale from 0 to 5 how often they exhibit the behaviour. The scales have been found to have reasonable internal consistency with alphas ranging from 0.65 to 0.86 (Oreyzi & Haghayegh, 2010) and has demonstrated evidence of construct validity in on-road behaviour (Zhao et al., 2012).

### Procedure

Participants completed the driving simulation, the MOA and the UFOV tasks. Participants either performed the driving simulation first or the MOA and UFOV tasks first and this was counterbalanced. The order in which the tasks were completed was counterbalanced for each participant. Breaks were given between each component of the study.

For the driving task, participants were instructed in how to use the driving simulator. This included how to use the steering wheel and pedals, the gears, the vehicle indicators etc. Participants were asked to follow UK road rules as they would when driving on real roads (stopping at red lights, giving way, maintaining lane positioning, etc). They were instructed they would be completing three designated routes and were to follow the auditory satellite navigation to navigate the route. This navigation comprised simple directional instructions such as “turn left at the next intersection”. These directions were given well in advance of having to make any manoeuvres. Eye-movement calibration was conducted before each route. The order in which the routes were completed were randomised for each participant.

For the UFOV, participants were instructed to identify, using a mouse, the object appearing in the middle of the screen (either a car or a truck) and, in the case of Subtasks 2 and 3, were asked to identify where the secondary target appears in the periphery. For the MOA task, participants were instructed to control the blue circle, with the mouse, and avoid the red circles that appear and move around the screen. They were told that the task would get increasingly harder as more circles were added on the screen and the trial would end if they collided with any red circle. Ten trials (two practice trials) were completed.

For the test–retest measure, participants were recalled 6 months after the initial testing phase to complete another MOA testing phase. The same MOA testing procedure as described above was used.

### Statistical design

Hierarchical linear regressions were conducted to investigate how MOA and UFOV performance predicted simulated driving performance and first fixation times (note, first time MOA scores were used and not test–retest scores). UFOV performance was initially entered into the models followed by MOA performance. Driving experience (years) was added into each model as a covariate. A paired samples *t* test and correlation were conducted to determine test–retest reliability after a 6-month period. Correlations were conducted to determine any relationships between the DQB subscales and MOA or UFOV.

## Results

All data and R scripts are available on the OSF. Link: https://osf.io/3gdcv/?view_only=d400ccc4769149fd9125300d8cb165ce

### Driving performance

An overall measure of driving performance was used here and was a point deduction system where a higher score suggests better driving performance. Driving performance was measured for each of the three courses separately and combined (as an average of the three routes). Driving experience was used as a covariate in these models. The relationship between driving experience and overall driving performance was not a linear relationship but rather a logarithmic relationship and therefore driving experience will be modelled as log transformed. A linear regression revealed that an increase in (log) driving experience predicted better overall driving performance (*F*(1,36) = 4.95, *R*^*2*^ = 0.1, *p* = 0.03). For the MOA task, individual trial performance was measured as the time (in seconds) until the participant-controlled blue circle collided with one of the hazard red circles. Ten trials were completed by participants with two initial practice trials. Performance was averaged across the remaining eight trials. To provide evidence that the number of trials used here is suitable to reliably measure actual performance, a one-way ANOVA was used to examine the variability in performance across the order of the trials. Performance across the trials was not significantly different overall (*F*(7,287) = 1.32, *p* = 0.24) or between Trial 1 and Trial 8 (*t*(41) = – 1.56, *p* = 0.13).

Participants’ scores for Subtask 3 in the UFOV were used (calculated by the software) where a lower value suggests better performance (faster speed of processing). Descriptive statistics for driving and task performance can be viewed in Table 1. Four participants did not complete the driving tasks in full, so their data was not used for the analyses involving driving scores. Two participants did not successfully finish the MOA task and one participant did not successfully complete the UFOV task, so their data were not used for any analyses.

**Table 1:** Descriptive statistics and correlations (*r* values) of driving, task performance and driving experience

Descriptive statistics						Correlations		
Task	*N*	Min	Max	M	SD	MOA	UFOV	Driving experience
Urban drive	38	5.52	9.55	8.12	0.94	0.5**	– 0.29	0.46**
Suburban drive	38	2.79	9.71	7.42	1.59	0.32*	– 0.15	0.18
Motorway drive	38	5.78	10	9.12	0.81	0.36*	– 0.19	0.35*
Overall	38	4.7	9.52	8.22	0.95	0.45**	– 0.23	0.35*
MOA (s)	42	11.7	42.79	27.76	7.35	-	– 0.14	0.57***
UFOV (ms)	41	15.1	153.1	48.71	30.44	– 0.14	-	– 0.28
Driving experience (years driving)	42	0.17	14	4.44	4.05	0.57***	– 0.28	-

*Significance at *p* < 0.05. **Significance at *p* < 0.01. ***Significance at *p* < 0.001. Correlations involving driving experience use log transformed driving experience

Table 1 highlights that MOA performance correlates with all driving tasks. Hierarchical linear regressions were conducted with performance on the different driving routes as outcome variables and UFOV and MOA performance as predictors. For each of the hierarchical linear regressions, the first model featured UFOV performance as a standalone predictor variable and (log) driving experience used a covariate (although this is merely a theoretical distinction; both act as predictors). The second model featured both UFOV and MOA performance as predictor variables, and (log) driving experience as a covariate.

Overall driving performance was not significantly predicted by UFOV performance and driving experience (*F*(2,34) = 2.64, *p* = 0.09, adjusted *R*^*2*^ = 0.08). When MOA performance was added to the model, the model significantly predicted overall driving performance (*F*(3,33) = 3.48, *p* = 0.027, adjusted *R*^*2*^ = 0.17). The difference between the first and second model was significant (*F*(1,33) = 4.61, *p* = 0.039). An increase in MOA performance predicted an increase in driving performance.

For the urban route, UFOV performance and driving experience significantly predicted driving performance (*F*(2,34) = 4.99, *p* = 0.013, adjusted *R*^*2*^ = 0.18). However, this was largely driven by driving experience rather than UFOV performance (Table 2). Adding MOA performance to the model improved the model fit (*F*(3,33) = 5.41, *p* = 0.004, adjusted *R*^*2*^ = 0.27). There was a significant difference between the two models (*F*(1,33) = 5.05, *p* = 0.031). An increase in MOA performance predicted an increase in driving performance.

**Table 2:** Summary of regression models

Outcome variable	Model	Predictor	*b*	Standard Error	*β*	*t*	*p*
Overall driving	1 (Driving experience (log) + UFOV)	Driving experience (log)	0.260	0.146	0.302	1.773	0.085
		UFOV	– 0.004	0.005	– 0.128	– 0.748	0.460
	2 (Driving experience (log) + UFOV + MOA)	Driving experience (log)	0.077	0.163	0.089	0.47	0.642
		UFOV	– 0.004	0.005	– 0.137	– 0.846	0.404
		MOA	0.051	0.024	0.387	2.147	0.039*
Urban drive	1 (Driving experience (log) + UFOV)	Driving experience (log)	0.343	0.137	0.402	2.498	0.018*
		UFOV	– 0.005	0.005	– 0.151	– 0.937	0.356
	2 (Driving experience (log) + UFOV + MOA)	Driving experience (log)	0.165	0.152	0.193	1.081	0.287
		UFOV	– 0.005	0.005	– 0.16	– 1.052	0.3
		MOA	0.049	0.022	0.381	2.248	0.031*
Suburban drive	1 (Driving experience (log) + UFOV)	Driving experience (log)	0.192	0.256	0.135	0.75	0.458
		UFOV	– 0.005	0.009	– 0.104	– 0.577	0.568
	2 (Driving experience (log) + UFOV + MOA)	Driving experience (log)	– 0.094	0.289	– 0.066	– 0.326	0.747
		UFOV	– 0.006	0.009	– 0.113	– 0.651	0.52
		MOA	0.079	0.042	0.366	1.896	0.067
Motorway drive	1 (Driving experience (log) + UFOV)	Driving experience (log)	0.244	0.122	0.34	1.999	0.054
		UFOV	– 0.002	0.004	– 0.078	– 0.429	0.67
	2 (Driving experience (log) + UFOV + MOA)	Driving experience (log)	0.16	0.142	0.222	1.122	0.27
		UFOV	– 0.002	0.004	– 0.08	– 0.462	0.647
		MOA	0.023	0.021	0.214	1.133	0.265

*Significance at *p* < 0.05

The models predicting driving performance for the suburban route were non-significant. This was the case when UFOV performance and driving experience were the predictors (*F*(2,34) = 0.68, *p* = 0.512, adjusted *R*^*2*^ = – 0.02), and when MOA performance was added as a predictor (*F*(3,33) = 1.69, *p* = 0.188, adjusted *R*^*2*^ = 0.05). The difference between the two models was non-significant (*F*(1,33) = 3.60, *p* = 0.067). The models predicting driving performance for the motorway route were also non-significant. This was found when UFOV performance and driving experience were predictors (*F*(2,34) = 2.73 *p* = 0.08, adjusted *R*^*2*^ = 0.09), and when MOA performance was added as a secondary predictor (*F*(3,33) = 2.26, *p* = 0.1, adjusted *R*^*2*^ = 0.1). There was no significant difference between the first and second model (*F*(1,33) = 1.28, *p* = 0.27).

#### Hazard perception (time to first fixate)

**Fig. 4:**
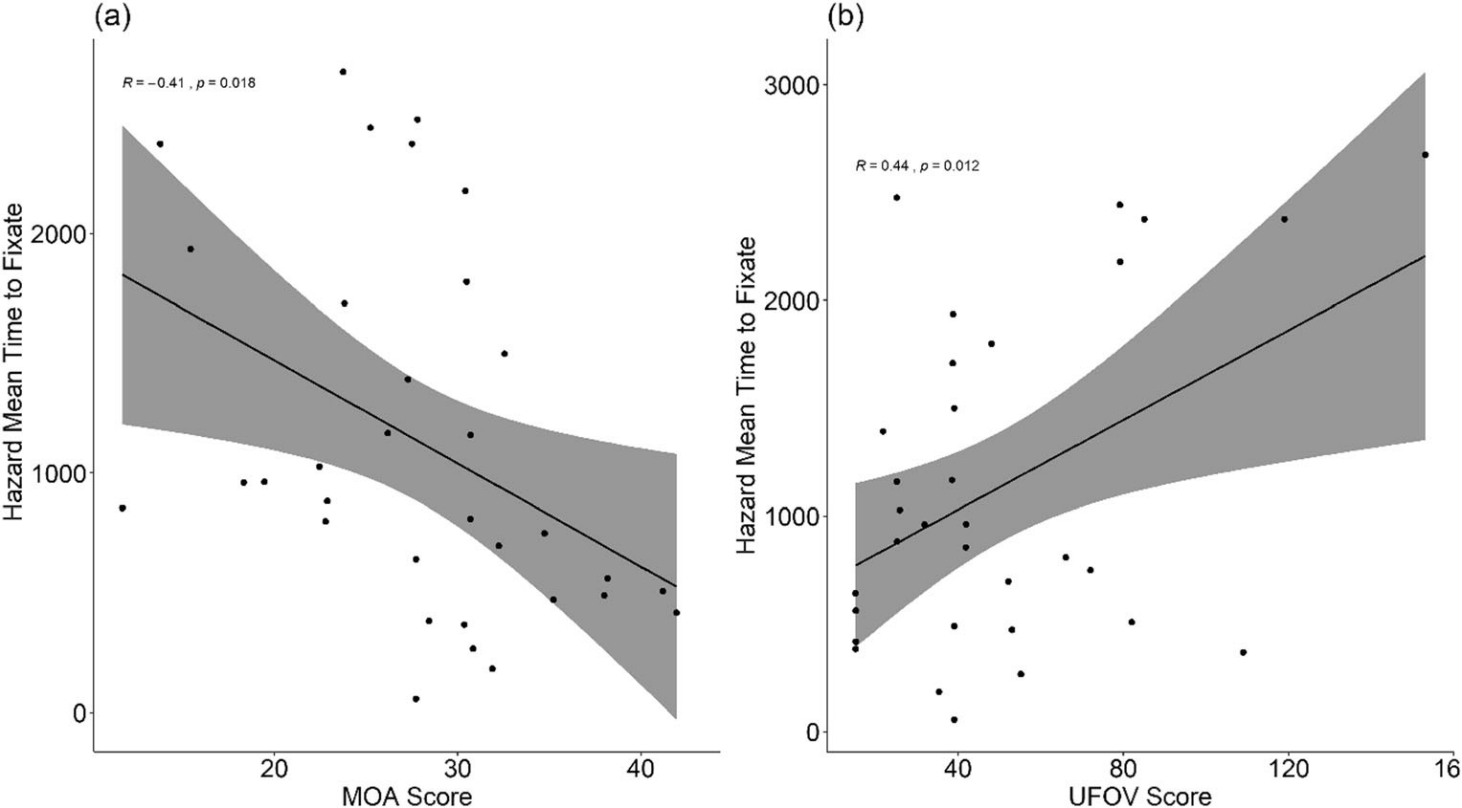
Relationship between post hazard onset time to fixate and scores in the MOA (**a**) and UFOV (**b**) task

Recorded eye movement data were analysed to examine the relationship between effective eye movements during hazard perception, and the two attention tasks. The time to fixate on hazards were calculated (TTF) for both the precursor and hazard stimuli. A Pearson’s correlation of TTF for precursor eye movements found no significant relationship when paired with either UFOV (*p* = 0.81*)* or MOA performance (*p* = 0.59). In comparison, when examining TTF for hazard onsets, a Pearson’s correlation was significant for MOA scores *R*(32) = – 0.41, *p* = .02 (Fig. 4a) and UFOV (*R*(32) = 0.44, *p* = 0.01 (Fig. 4b). Better performance in these tasks correlated with faster detection of hazards. There was also a significant relationship between (log) Driving Experience and hazard fixation times with increased driving experience correlating with faster detection times (*R*(32) = – 0.54, *p* = 0.001)

To further investigate the relationships between effective eye behaviour (TTF performance) and UFOV and MOA performance, hierarchical regressions were conducted with TTF for the hazard onset period as the outcome variable. As in the hierarchical regressions of driving performance, the UFOV scores were entered as a single predictor in the first model with (log) driving expertise a covariate and then with MOA performance added as a secondary predictor in the second model. In the first model, UFOV performance and driving experience predicted TTF (*F*(2,29) = 8.31, *p* = 0.001, adjusted *R*^*2*^ = 0.32); (UFOV: *β* = 0.29, *t* = 1.88, *p* = 0.07); (Driving experience: *β* = – 0.44, *t* = – 2.80, *p* = 0.01).

TTF performance was also significantly predicted when MOA performance was added as a second predictor along with UFOV performance and driving experience (*F*(3,28) = 6.30, *p* = 0.002, adjusted *R*^*2*^ = 0.34). There was no significant difference between the two models (*F*(1,28) = 0.82, *p* = 0.19). In the second model, UFOV (*β* = 0.30, *t* = 1.96, *p* = 0.06), MOA (*β* = – 0.23, *t* = – 1.35, *p* = 0.19), and driving experience (*β* = – 0.32, *t* = – 1.84, *p* = 0.08) were not significant predictors.

### Test–retest reliability

For both test stages of the MOA task, performance was measured by the time (seconds) in which the target object collided with another object across eight trials. Descriptive statistics of the performance across the two tasks are shown in Table 3. Performance across trials during did not significantly differ overall *F*(7,216) = 0.88, *p* = 0.52), or between Trial 1 and Trial 8 (*t*(27) = – 0.43, *p* = 0.67 suggesting no evidence for general improvement across trials within the testing session.

**Table 3:** Descriptive statistics for the Multiple-Object Avoidance (MOA) at both time points

*N*	Mean age (SD)	Mean years of driving experience (SD)	Minimum	Maximum	Median	Mean	SD
28	24.39 (*4.56*)	5.41 (4.52)	13.77	42.79	29.51	28.73	6.88
28			18.15	47	29.92	30.47	7.93

**Fig. 5:**
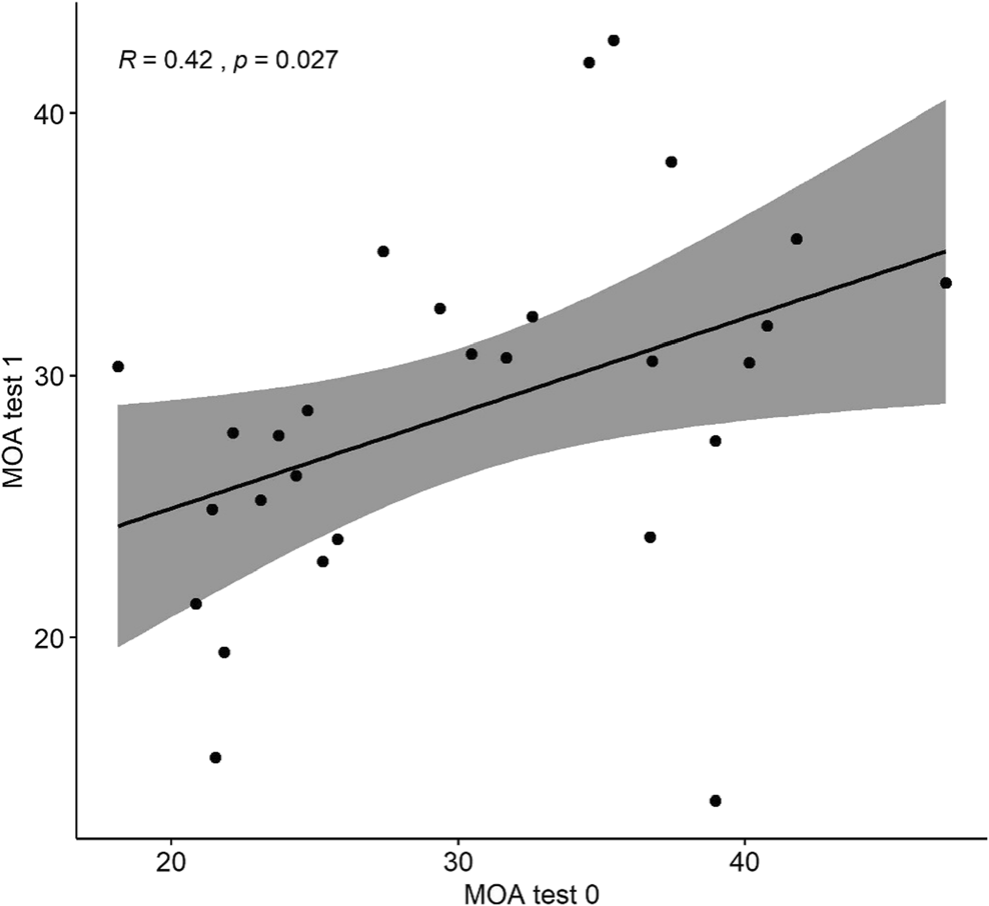
Relationship between performance in the MOA task at MOA0 and MOA1

Two methods of analysis were employed to assess the consistency and reliability of the MOA task over time. Paired *t* tests were used to examine the difference in mean response time over time. There was no significant difference between MOA0 and MOA1 performance, *t*(27) = 1.15, *p* = 0.26. Pearson’s correlation coefficients were calculated to examine the reliability of individual subjects across both samples. Performance between the two tasks were positively correlated, *r*(27) = 0.42, *p* = 0.027. Figure 5 shows the relationship of MOA performance between MOA0 and MOA1.

### Relationship with Driving Behaviour Questionnaire

MOA performance correlated with the *Lapses* subscale (*r* = – 0.35, *p* = 0.03). As performance in MOA increased the number of instances of attentional lapses decreased. There was no relationship between MOA performance and the number of *Errors* (r = – 0.21, *p* = 0.2), the number of *Aggressive Violations* (*r* = 0.06, *p* = 0.73) and the number of *Ordinary Violations* (*r* = – 0.12, *p* = 0.47). UFOV performance correlated with the *Ordinary Violations* (*r* = – 0.45, *p* = 0.006) and weakly with the *Errors* subscale (*r* = – 0.33, *p* = 0.05). Interestingly, as performance in the UFOV task decreased (slower processing speed) the number of instances of *Ordinary Violations* and *Errors* also decreased. There was no relationship for the *Lapses* subscale (*r* = – 0.31, *p* = 0.06), and the *Aggressive Violations subscale* (*r* = – 0.3, *p* = 0.07).

## Discussion

The overall aim of this study was to replicate and expand on Mackenzie and Harris' (2017) exploration of the relationship between MOA performance and driving behaviour. The first aim was to replicate previous findings and investigate how well MOA performance predicted simulated driving performance and compare this to the predictive power of the UFOV task. We showed that the MOA does well in predicting driving performance, particularly in complex environments and does better than the UFOV at predicting driving performance. The second aim was to investigate how well the MOA predicts hazard perception; as measured by first fixation eye movements (TTF). We hypothesised MOA performance would more strongly correlate to first fixation times than the UFOV task given the active nature of the MOA. We found a relationship between MOA performance and TTF, but this was more robust for UFOV. The third aim was to investigate MOA task test-re-test reliability and explore its convergent validity with a self-report driving measure of visual attention when driving. We observed the hypothesised correlation in performance between testing and re-testing stages; suggesting reliability of this test to measure attentional function. We also found evidence of convergent validity with the subscale of the Driving Behaviour Questionnaire that specifically measures attentional lapses.

### The MOA, attention, driving performance and hazard perception

We find here that attentional function, as measured by the MOA task, significantly predicted simulated driving performance, replicating the previous result of Mackenzie and Harris (2017). This is line with other studies that demonstrate the relationship between superior attentional function as measured by reduced attention tasks and improved driving performance (Karimi et al., 2015; Owsley & McGwin Jr., 2010; Roca et al., 2013; Weaver et al., 2009; Wood et al., 2016). If one demonstrates superior attentional function in a reduced task, then it seems unsurprising this would extend to a more complex task that may also involve these attentional components to a degree given what we know about cognitive transfer (Peng & Miller, 2016; Posner et al., 2015). If one is better able to, for example, divide attention across task operations such as vehicle control and identifying potential hazards, sustain their attention to the important aspects of the driving environment and effectively ignore irrelevant stimuli, they likely would be a better driver.

Previous research has discussed how some experimentally-reduced tasks such as the UFOV and ANT measure only properties of attention to brief stimulus presentation (Bowers et al., 2011; Mackenzie & Harris, 2017) where stimuli are presented for up to several hundred milliseconds. Tasks such as Multiple Object Tracking, which provide a measure of sustained divided attention to dynamic stimuli, are arguably more representative of driving due to their more temporally extended nature. We go further with the MOA where we argue that it also represents the more active visual attentional function involved in driving. In the MOA, one must actively be in visuomotor control of an object; a task we suggest targets the more active visual elements of everyday tasks (see Hommel, 2010; Humphreys et al., 2010; Land, 2006; Land & Lee, 1994; Mackenzie & Harris, 2015; Tatler et al., 2011). In addition, in MOA as in driving, one must divide attention to relevant stimuli, but the relevant importance of the objects changes in real-time during the MOA task. Only those objects that are either near or judged to potentially collide with the user’s object are directly relevant. This divided attention involved in controlling an object and predicting the behaviour (e.g. motion) of other objects could possibly be analogous to controlling a vehicle while predicting potentially hazardous events. The similarities in attentional processing between MOA and driving may explain the predictive power of the MOA task here. Supporting this claim may be the finding that this attention task correlates with the attentional *lapses* subscale on the DQB, suggesting convergent validity in the MOA’s ability to measure attentional function in relation to driving performance.

It is important to note that, whilst performance on the MOA predicted overall general driving performance, this was largely driven by driving performance during the most complex environment. As the complexity of the driving environment decreased, so did the predictive power of MOA performance. This is perhaps unsurprising however given the routes used in this study. This finding indeed mimics the eye movement finding in Mackenzie and Harris (2017) where MOA performance predicted increased visual scanning during more complex drives. The complex urban environment would demand more attentional resources in order to, for example, detect and respond to pedestrians walking across the road, searching intersections before committing to cross, turning across traffic (right turn in the UK) etc. In comparison to a simple and straight two-lane motorway where traffic is more regular and (arguably) more predictable; where maintaining lane positing may be more of the priority in order to drive successfully. As such, it would make sense for a relatively demanding visual attention task such as the MOA to predict performance during a drive that would place more of a demand on visual attention function.

Concerning the relationship between MOA performance and hazard perception we find some evidence that better performance was related to earlier hazard fixation times (TTF). The MOA requires one to make many eye movements; but mainly eye movements to open space to which the users’ target will imminently be moved, and to the hazard circles. This type of top-down intentional eye movement behaviour may mimic one looking ahead to where they are manoeuvring the vehicle and searching for hazards on the road, and thus, may explain the link we find here. However, the evidence for a strong predictive relationship was not observed when UFOV performance is included. UFOV performance had a stronger relationship to TTF than MOA (albeit not significant in the full regression model). The responsive nature rather than the predictive nature of the hazards and the speed of visual processing nature of the UFOV may explain this effect. There was no evidence that task performance (either MOA or UFOV) predicted fixations towards hazard precursors. In other words, participants were able to make similarly timed eye movements to the pre-onset hazards irrespective of visual attentional function. It was only after the pedestrians began to step into the road (and thus became fully developed hazards) that UFOV performance better related to fixation times. If an individual has faster visual processing speed (as measured by UFOV) then this may better allow them to make a re-fixation to the hazard when they acknowledge that the pedestrian is now a fully developed hazard. Whilst there could be an element of visual processing speed in the MOA, arguably this is better measured by the UFOV.

However, the predictive effect was not strong for either task. It would be of interest to investigate the relationship between MOA ability and the ability to make fixations to less subtle hazards, e.g. environmental hazards (Crundall et al., 2012; Shahar et al., 2010). We also highlight the limitation in our measure of TTF. Future work could investigate the relationship between MOA performance and the visual processing of a range of hazard types, where, for example fixation durations might offer more sensitive insights into hazard processing. For example, they may offer insights into processing times and indeed offer insights where longer processing might result in subsequent inattention to other relevant environmental cues (Velichkovsky et al., 2002). One might argue, however, that hazard fixations are not a good measure of hazard perception at all. It would be interesting to correlate MOA performance on more sensitive measures of hazard perception, or specifically, hazard prediction, such as the What Happens Next test (Crundall, 2016; Jackson et al., 2009) – a task that arguably better capture the ability to understand hazardous situations.

In summary, Study 1 has measured the ability of MOA task performance to predict simulated driving performance relative to the UFOV. It does this well for complex driving environments and the results highlight the potential for future work to be carried out in a number of further areas such as hazard prediction. Study 2 now investigates the MOA’s ability to measure individual differences in visual attention function in relation to sporting expertise whilst also investigating elements of construct validity raised in Study 1 by investigating MOAs relationship to cognitive processing speed and object tracking.

## Study 2: The MOA in sport and its potential attentional composition

The MOA was initially developed to measure visual attention function in relation to driving behaviour. As with Multiple Object Tracking (MOT) tasks, the use of the MOA to investigate visual attention function in other ‘everyday’ tasks might be attractive to researchers given the attentional similarities between these tracking tasks. In this study, we therefore aim to investigate how the MOA might discriminate visual attentional function between individuals who play sports and individuals who do not whilst also investigating its possible attentional relatedness to other tasks often reported as being important in sporting performance: object tracking and cognitive processing speed.

### Individual differences in visual attention in sport

The ability to control attention is important during complex activity such as sport (Engle, 2002). In many “strategic” sports (Voss et al., 2010), such as football or basketball, environments are typically visually noisy and require individuals to attend to multiple objects, inhibit task-irrelevant information, store object location information and make fast decisions. One might argue that an individual who engages in these types of sport might therefore exhibit superior attentional function in these domains or, indeed, vice versa where individuals who have superior attentional function exhibit superior sporting performance. There is mounting evidence for individual differences in lab-based measures of visual attentional control – both within sporting experience (i.e. high and low skilled players) and across sporting experience (sports players and non-sports players). For example, working memory capacity in basketball players (Furley & Memmert, 2012), temporal processing in tennis players (Overney et al., 2008), attentional window in soccer players (Scharfen & Memmert, 2019) and inattentional blindness in basketball players (Furley et al., 2010).

In their meta-analysis, Voss et al., (2010) report a medium effect for differences in processing speed (measured in a number of ways) across the literature. Arguably, fast processing times are needed for most sports players in order to react and make decisions in real-time. Importantly however, sporting type seemed to drive the differences between sporting and non-sporting individuals. The effect was stronger for what was termed “interceptive sports” (a sport that requires coordination between the body or a held implement and an object in the environment, e.g. badminton) compared to strategic sports (a sport that usually involves varied situations and requires the simultaneous processing of information regarding teammate, opponents, ball position etc., e.g. football).

Concerning object tracking and divided attention, similar results are observed. Howard et al., (2018) showed that those who engaged in team ball sport showed superior performance in a modified MOT task as well as a rapid visual presentation task, with both tasks requiring sustained attention to rapidly changing stimuli. Similarly, Qiu et al., (2018) report that elite basketball players outperformed intermediate and non-athletes during a MOT type task. Furthermore, Harris et al., (2020) found a similar result in football and rugby players. They argue this expertise is linked with an increased processing capacity rather than a more effective perceptual-cognitive strategy. In explaining the differences in attentional processing between sporting and non-sporting individuals in general, one might favour the “deliberate practice” view (e.g. Ericsson et al., 1993) where one obtains expertise through effortful and continuous engagement in a task. In other words, if one continually engages in tasks that target divided attention mechanisms or require fast processing speed, one improves in this attentional domain. However, see Hambrick et al., (2016) for a detailed discussion on the possible direction of causality. Irrespective of current debates in the field, with these individual differences described above, one could hypothesise the MOA might allow us to discern differences in visual attention function between those with different levels of sporting expertise. We test this here and aim to investigate any differences in MOA performance between individuals who play interoceptive/strategic sports and those that do not. Given the individual differences in attentional performance described above, we hypothesised that individuals who played sport would outperform those who do not.

In addition, we have suggested, in Study 1, that the two measures of visual attention function discussed above that are important in sport (object tracking and processing speed), might be attentional components involved in MOA performance, and we have offered these as explanations that might explain the efficacy of MOA in predicting driving behaviour and hazard perception performance. To provide some support to these claims, we used Study 2 as an opportunity to also investigate the relationship between these individual measures of attention and MOA performance. This would help to provide evidence of the proposed attentional composition of the MOA – providing some evidence of construct validity – but also aids in establishing the task beyond driving as these proposed attentional components are likely to be involved sport. This study aimed therefore to also investigate the contribution of sustained divided attention and inhibitory control (as assessed by the MOT task) and cognitive processing speed (assessed by a digit symbol substitution task) on MOA performance. This was investigated statistically in a hierarchical regression by examining the variability of MOA performance that can be explained by cognitive processing speed and object tracking ability. We hypothesised that MOA performance should positively correlate with MOT performance, given previous findings and the similar nature of spatiotemporal tracking involved, and also positively correlate with DSST performance, given the visually demanding nature of the MOA.

## Methods

### Participants

Forty-seven participants took part in this study (17 males; mean = 28.4; SD = 10.6). A sample calculation was conducted in *R* using the package *pwr (v.1.3-0)*. Effect size was predicted using recent sport and MOT research showing performance differences in sporting group with an effect size of *η*_*p*_^*2*^ = 0.163 (Harris et al., 2020). With a conservative estimate of power at 0.95, alpha error probability of 0.05 in a two-independent samples comparison test, 34 participants would be required in each group. Again, we report we are underpowered in obtaining this effect size for this study. A lower estimate of power at 0.80, alpha error probability of 0.05 would suggest 21 participants be recruited in each group for this effect size. This group of participants was split into two groups. The sports group comprised of 21 participants (12 males) with a mean age of 24 (SD = 4.41). They had all played sports competitively (for a club) for a minimum of 1 year and within a sport that was either “strategic” or “interoceptive” in nature (as loosely defined in Section 3.1.2.). They were currently still competing/playing at the point of testing. The list of sports included: football, rugby, basketball, netball, martial arts, badminton and slalom canoe. The non-sport group had 26 participants (five males) with a mean age of 31.9 (SD = 12.8). All participants declared they do not take part in competitive sport. Participants received an Amazon Voucher worth £10 for participating.

### Materials

#### Multiple Object Avoidance (MOA) task

*See Study 1 for details on MOA procedure (2.2.2.1)*.

#### Multiple Object Tracking (MOT) task

**Fig. 6:**
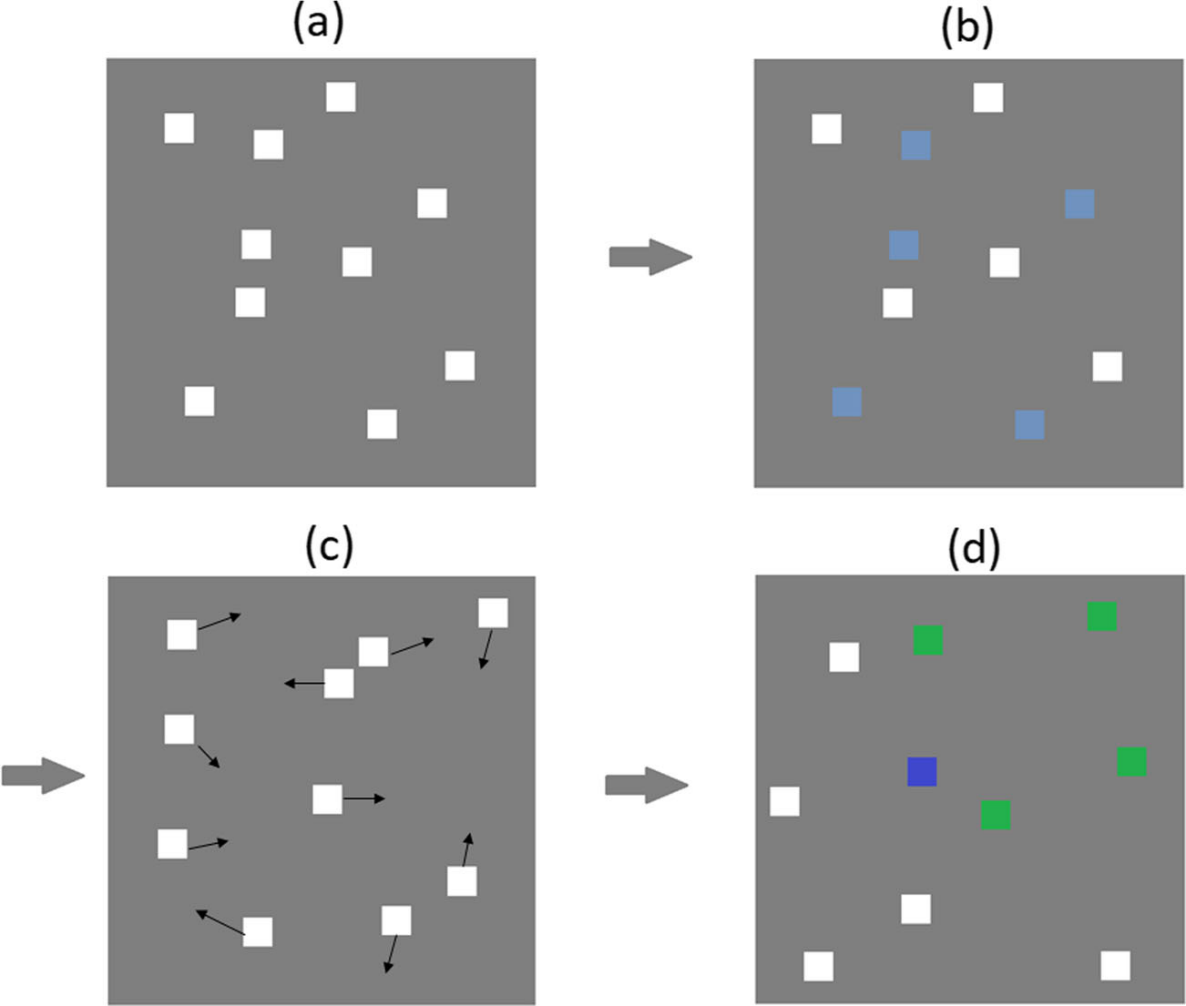
The MOT task. Participants are presented with ten squares (**a**). Five target squares begin to flash (**b**) before stopping and all squares begin moving around (**c**). Participants then click on the five squares they believe to be the original ones that flashed. In this example (**d**), the participant has got 4 out of 5 correct

This task was programmed using VisionEgg. Initially, ten squares are presented to participants, each 30 by 30 pixels in size (~7.9 by 7.9mm) in a random position on the screen within a window of 1014 by 758 pixels. Five randomly selected squares begin to flash, and it is the participant’s task to attend to these five squares while ignoring the other five. They will stop flashing and all squares begin to move around the screen in a vector-like fashion. Each square will move in one of twelve randomly assigned angular directions (18, 45, 72, 108, 135, 162, 198, 225, 252, 288, 315, 342 degrees). They move at a randomly assigned speed of either 60, 134 or 180 pixels per second. Squares can overlap with each other during this movement sequence. All squares will stop, and participants must click on the five squares they identified as being the original five squares that flashed (see Fig. 6 for a sequential representation). Participants completed a total of 30 trials. A mean accuracy across these 30 trials was calculated as MOT performance. The number of trials used was broadly comparable to previous MOT research (Howard et al., 2017; Howard & Holcombe, 2008; Mackenzie & Harris, 2017). Given the more ordinal measure of performance per trial in this type of MOT task, where a participant could only receive a score of 0, 20%, 40%, 60%, 80% or 100%, a larger number of trials are required to obtain suitable variability across participants.

The task was presented on a 17.5-inch CTX EX951F monitor (Chuntex Electronic Co., Ltd., Taipei, Taiwan) with a refresh rate of 85 Hz and at a screen resolution of 1280 x 1024.

#### Digit Symbol Substitution Task (DSST)

**Fig. 7:**
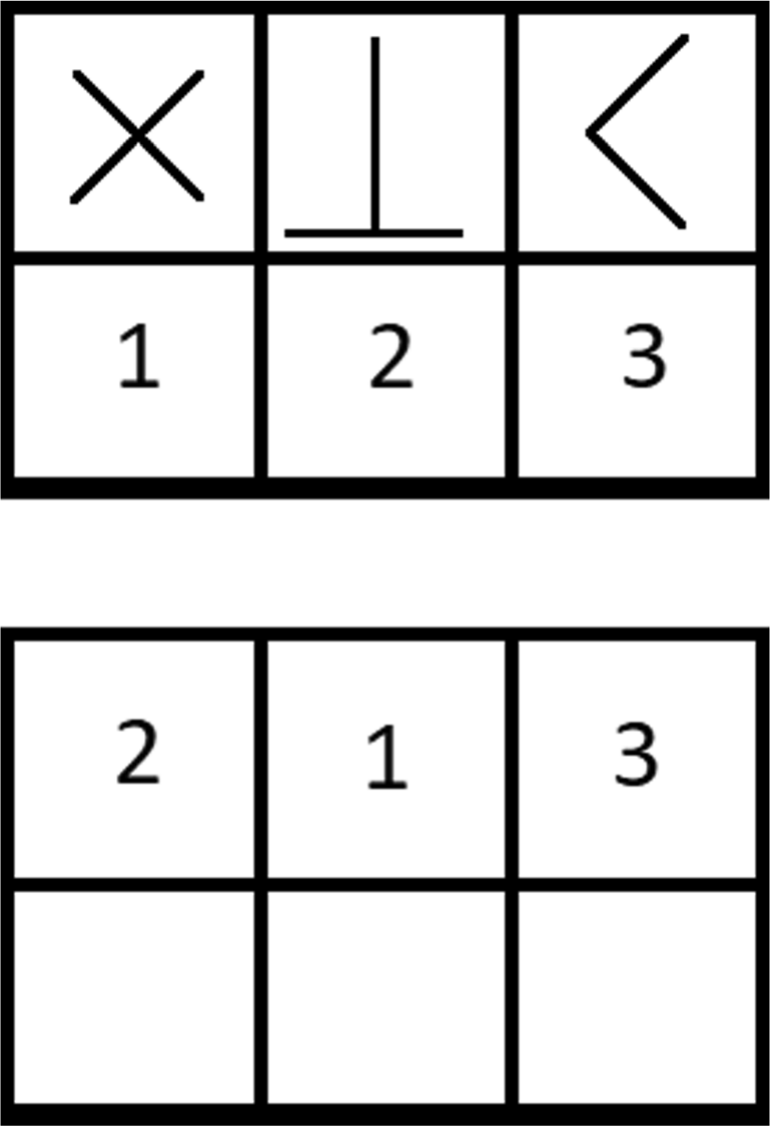
Example representation, using three symbols, of the DSST. Participants are presented with a list of nine symbols with a corresponding number. They then have 60 s to fill in the blank field with the corresponding symbol

Using a similar method to the original DSST task (Wechsler, 1981), participants are presented with a sheet of paper with a symbol key of nine symbols that are attached to a number. Below this, there is an array of blank boxes with a number above (Fig. 7). They have 60 s to write the corresponding symbol using the symbol key in the appropriate blank box. Participants must fill as many boxes within 60 s using a pen. The total number of correctly written symbols is recorded as DSST performance. There is a maximum of 93 boxes that can be filled.

### Statistical design

Correlations were initially conducted to determine any relationship between the three tasks. A hierarchical regression was conducted to determine the variability of MOA performance that is explained by first MOT performance and then DSST performance. Age was used as a covariate in the regression analyses to account for any age-related effects of attentional function in these tasks.

Linear models were used to determine the variability of task performance that can be explained by sporting group (sport and non-sports). A multivariate linear model was conducted to determine if overall visual attentional function (as measured by the three tasks) could be predicted by sporting Group (sports and non-sports). Individual linear models for each task are reported too. Age significantly differed between the two sporting groups (*t*(45) = 2.68, *p* = 0.01), with the non-sports being older (*M* = 31.88) than the sports group (*M* = 24.05). Thus, the variability explained by age was accounted for in all analyses as a covariate.

### Procedure

Participants were asked to conduct the three different cognitive tasks as described above. All tasks were completed in a randomised order across participants. Ethical approval was given by Nottingham Trent University College Research Ethics Committee.

## Results

### Sporting expertise and task performance

For the MOA task, individual trial performance was measured as the time (in seconds) until the participant-controlled blue circle collided with one of the hazard red circles. Ten trials were completed by participants with two initial practice trials. Performance was averaged across the remaining eight trials. For the MOT task, trial performance was calculated as a percentage of correctly identified objects out of a total of five. This was averaged across 30 trials. A larger number of trials are required for MOT to provide variability in scores across participants. Given the data are percentages, these data were appropriately transformed using a logit function for the analyses. DSST score was recorded as the total number of symbols corrected substituted within 1 min.

**Fig. 8:**
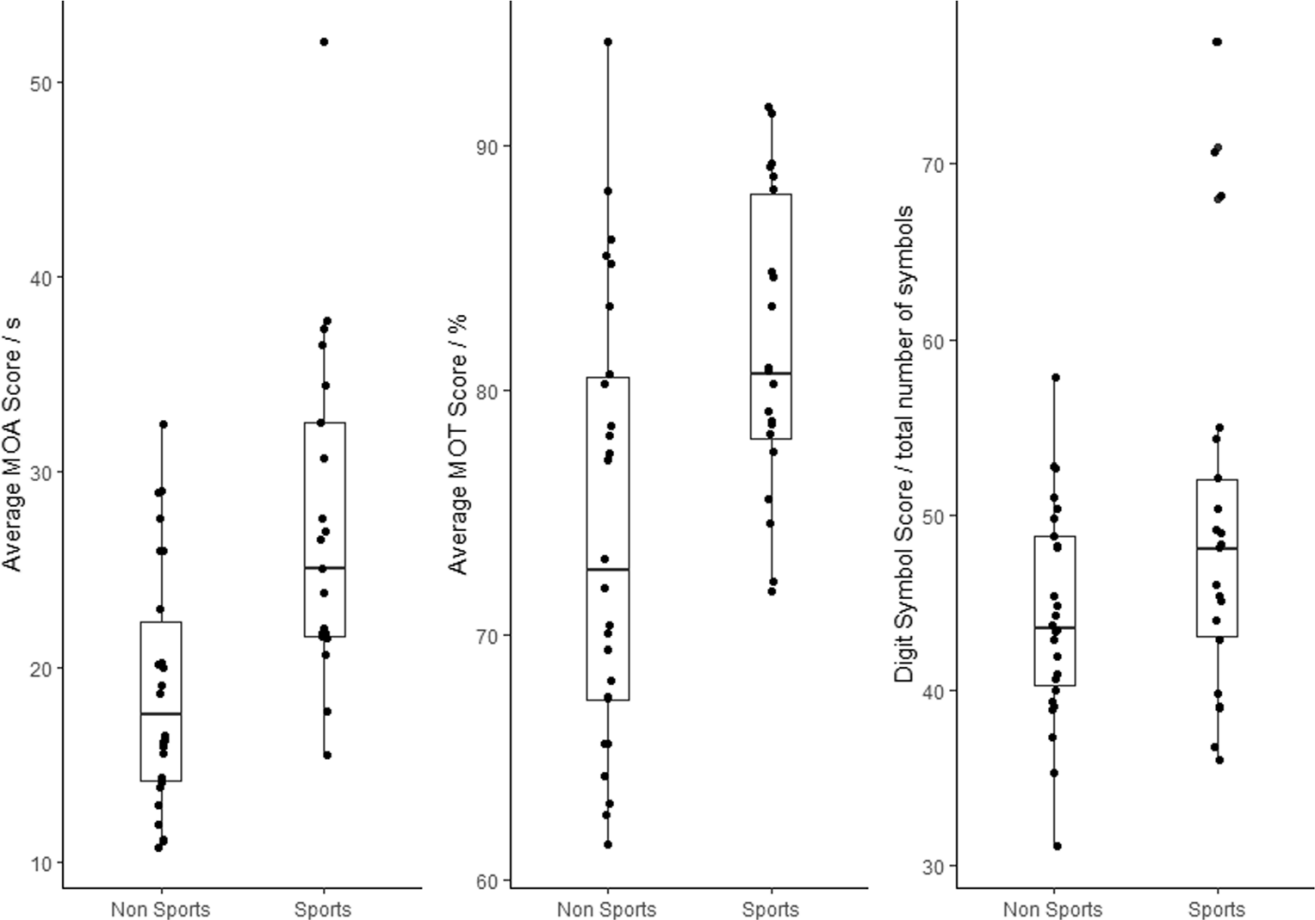
Descriptive statistics of performance for each of the three tasks per sporting group. Data show all data points, maximum, minimum, median and interquartile data points

Task performance was compared across sporting groups. Age was included as a covariate throughout. The multivariate model revealed that Sporting Group predicted attentional function where those in the sporting group had superior visual attentional function as measured by the three tasks combined (*V* = 0.24, *F*(3,42) = 4.5, *p* = 0.008). Individually, those in the sport group performed better in the MOA task (*b* = 7.82, SE = 2.38, *t* = 3.28, *p* = 0.002) and the MOT task (*b* = 0.38, SE = 0.16, *t* = 2.32, *p* = 0.025) and in the DSST (*b* = 5.84, SE = 2.74, *t* = 2.13, *p* = 0.039) (Fig. 8).

### Relationship between MOA, MOT and processing speed

Descriptive statistics for task performance can be viewed in Table 4. Pearson correlations were conducted to determine any simple relationships in performance between these tasks. All tasks were significantly positively correlated with each other (Table 4).

**Table 4:** Descriptive statistics and correlations (*r* values) of task performance. Note: MOA is measured in seconds, MOT is measured as a percentage and DSST is measured as a total number

Descriptive Statistics						Correlations	
Attention task	*N*	Min	Max	M	SD	MOA	MOT
MOA	47	10.7	52.1	22.6	8.49	-	
MOT	47	61.3	94	77.7	8.65	0.32*	-
DSST	47	31	77	46.5	8.90	0.48**	0.48**

*Significance at *p* < 0.05. **Significance at *p* < 0.01

To investigate the relationship between these tasks and the shared variability further, a hierarchical regression was conducted with MOA performance as the outcome variable. Age was used as a covariate in these models. MOT scores were entered into the model first given previous research (note: Logit MOT scores are used for regression analyses). The model significantly predicted MOA performance (*F*(2,44) = 3.45, *p* = 0.04, adjusted *R*^*2*^ = 0.1). MOT performance weakly and non-significantly predicted MOA performance (*b* = 4.29, *t* = 1.92, *p* = 0.06). When DSST scores were added into the model, the model significantly predicted MOA performance (*F*(3,43) = 6.11, *p* = 0.001, adjusted *R*^*2*^ = 0.25). The difference between the first and second model was significant (*F* (1,43) = 10.03, *p* = 0.003). See Table 5 for individual predictor coefficients for each model. From this table, it is evident that whilst MOT is weakly related to MOA in the first model, the variability of MOA performance that is accounted by MOT performance can likely be explained by cognitive processing speed in the second model (Table 5).

**Table 5:** Overview of regression models

Model	Predictor	*b*	Standard error	*β*	*t*	*p*
1	MOT	4.29	2.23	-	1.92	0.06
2	MOT	0.67	2.33	0.04	0.29	0.77
	DSST	0.44	0.14	0.46	3.17	< 0.01

## Discussion

The aims in this study were two-fold. The first was to identify if the MOA task was able to discriminate the visual attentional function of between individuals who play interoceptive/strategic sports and those that do not. We predicted that those who played sports would show superior visual attentional function as measured by the MOA task. We found evidence to support this and also observed superior performance by the sports players in the multiple object tracking MOT task and Cognitive Processing speed task (DSST), replicating previous research (Howard et al., 2018; Qiu et al., 2018). The second aim was to investigate the relationship between MOA performance and the two measures of speed of cognitive processing and attentional tracking. We hypothesised that higher scores in both the DSST and MOT would predict higher scores in the MOA task. There was some evidence of this where both tasks correlated with the MOA but the DSST was the stronger and only predictor of MOA performance with much of the variability of MOA performance initially explained by MOT accounted for by DSST. The results are discussed in turn.

### Sporting expertise and task performance

Previous research has indicated that there are individual differences in attentional performance between those with different levels of sporting expertise within specific attentional domains such as working memory (Furley & Wood, 2016), processing speed (Voss et al., 2010) or divided attention (Harris et al., 2020), with superior sports players often exhibiting superior attentional function. With regards MOA, whilst we are not able to identify a single attentional mechanism, we find a similar result with sports players performing better than the non-sporting individuals. This may be due to similar attentional mechanisms involved in both sport and MOA, e.g. divided attention, level of processing speed, visuomotor control. In this study, our sporting participants played strategic sports (i.e. where environments are typically visually noisy, requires continually tracking of multiple objects, inhibit task-irrelevant information, make quick real-time decisions) or interoceptive (i.e. when one requires coordination of an object in the environment) which arguably incorporates the hypothesised attentional properties of MOA. In line with the deliberate-practice literature (e.g. Ericsson et al., 1993), athletes with experience in a specific sport may only exhibit attentional expertise in domains specific to that sport (Furley & Wood, 2016), which is why we might see superior performance in MOA given its arguable similarity to the attention required in these sports.

One further and more specific possible attentional mechanism that might explain the link between attention-in-sport-expertise and MOA performance might come from the biased competition theory (BCT) (Desimone & Duncan, 1995). This model of visual attention proposes that attentional selection is competition based. Bottom-up mechanisms isolate the individual competing objects based on features and top-down attentional mechanisms select specific objects that are deemed behaviourally relevant at that time. Importantly, the representation of what is deemed behaviourally relevant is stored in working memory (Downing, 2000; Huang & Pashler, 2007). The same neural mechanisms that are explained by the BCT might be involved during MOA. All objects on screen will attract attention but only those that are behaviourally relevant – those that may result in a collision – will be attended to and subsequently acted on i.e. moved away from. This type of top-down competitive attentional selection was investigated in the context of (simulated) sport where Furley and Memmert (2013) report that basketball players showed an advantage in making decisions regarding whom they should pass to when they had previous access to the visual information of the relevant player that they consequently stored in their working memory. These attentional mechanisms involved in sport and MOA are arguably similar and thus may explain the relationship we observe here with sporting individuals exhibiting superior MOA performance. The relationship between working memory function is key here but its role in MOA, whilst hypothesised, has not yet been established. Future research might investigate the relationship between working memory further and MOA further.

Although, importantly there are often differences in attention control across sports. Overney et al., (2008) found that not only did tennis players outperform non-athletes, but they outperformed triathletes too in measures of temporal processing. Similarly, Meng et al. (2019) found that volleyball players performed better than badminton players on some tasks such as attentional alerting, whereas the badminton players performed better than volleyball players on measures such as processing speed. Future research might aim to investigate individual differences in visual attention function as measured by the MOA in further defined sporting categories in order to further our understanding of the attentional nature of the MOA.

### Attentional similarity between MOA, MOT and cognitive processing speed

The initial correlational relationship found between MOT and MOA is perhaps unsurprising and we replicate this finding from before (Mackenzie & Harris, 2017). Both tasks are object tracking-based that require one to divide attention to multiple and dynamic stimuli. However, with adding the interactive visuomotor control element, the attentional nature of the MOA task deviates to some extent from that of MOT. Unlike the MOT which requires no manual interaction and may not always be expected to engage even oculomotor interaction (Fehd & Seiffert, 2008; Oksama & Hyönä, 2016; Zelinsky & Neider, 2008), the MOA might require more eye movements in order to visually guide the blue circle given what we know about the link between vision and guided action (Hayhoe & Ballard, 2005; Land, 2006; Land & Hayhoe, 2001; Pelz et al., 2001). In addition, unlike in the MOT where the status of each object as a target or non-target is constant within a trial, the relative importance of each object to track in the MOA changes substantially based on its perceived likelihood of collision. Furthermore, the MOA requires participants to predict movement and also to constantly react to movement. If, for example, a red circle collides with another red circle, this will change its initial trajectory and the participant may need to react in order to move their blue circle. These differences may begin to explain the weak relationship between MOT and MOA performance.

With these attentional demands of the MOA, it is arguably unsurprising that we see a much stronger relationship between MOA and cognitive processing speed (at least as measured by DSST). The DSST is also quite active in nature, requires planning and executive control and, albeit to a lesser extent, to divide attention across elements. As the term suggests, cognitive processing speed can loosely be defined as the duration required by an individual to process specific information in order to act or make a decision (Owsley, 2013). Processing speed has been found to correlate with cognitive functions such as working memory (Salthouse, 1992), executive function (Salthouse, 2005) and intelligence (Fry & Hale, 2000). Visual processing speed specifically is likely relevant in the MOA given the demanding visual nature of the dynamic task. As the “hazard” circles move around, and not at a particularly slow speed, one must be able to monitor the ever-changing movement of the circles, predict their movement and, importantly, be ready to respond if a collision with a hazard circle is imminent by moving the controlled circle out of the way. This all requires the ability to process a constantly increasing amount of visual information as the number of hazard circles increases.

## General discussion

Our overall aim was to highlight the Multiple Object Avoidance task and investigate its predictive value in assessing attention for action by using the cases of driving and sport. Two studies have provided evidence for these. Study 1 showed that MOA performance predicts driving performance and is associated with hazard perception performance in a simulated driving environment. It also found the possible utility in predicting driving behaviour compared to other commonly cited tasks in the UFOV literature. Study 2 showed that MOA performance is discriminative between those who plays sports and those who do not; providing evidence for its sensitivity to individual differences in visual attentional function, and showed that MOA performance appears to be related to cognitive processing speed and, to a lesser extent, Multiple Object Tracking skill.

Overall, the MOA task appears to measure individual differences in general visual attentional function within everyday settings with an interactive visuomotor component. Importantly, in driving, these findings appear in a young adult population and cannot therefore be due to the age-related effects in cognitive decline that can account for findings in much of the literature (see Bédard et al., 2016; Liebherr et al., 2019). Both driving and sport rely on complex interaction with the environment and attentional components such as divided attention, tracking, high speed processing and executive control. We suggest these processes are captured within the MOA task and may drive the relationships we find here.

However, the correlational nature of the research presented here does not allow us to identify a causal relationship and only a true experimental manipulation will be able to do this. In sports, for example, it is certainly far from clear in this research if one has developed visual attention expertise through playing sport and thus performs better at the MOA, or if one exhibits visual attention expertise (that could be measured by the MOA) and therefore be able to demonstrate expertise in sport. Indeed, In the case of driving here, when taking the reverse of the regression model reported, we would see that driving experience predicted MOA performance. This suggests that one could train the attentional components in the real-world task that is then represented or measured in the MOA. There are views that cognitive skills are acquired through practice that increases efficiency of processing (e.g. Eccles, 2006) or that expertise in tasks are explained by individual performance in certain domains (e.g. Hambrick et al., 2016) but investigating this within MOA and the relation to everyday tasks is beyond the scope of this research. This would certainly merit further investigation using the MOA. Indeed, using MOA as a measure of visual attention function may inform this debate.

We also suggest that the MOA captures individual differences in visual attention within these everyday tasks because it shares some attentional processing requirements involved in these tasks. We have provided some evidence for this in the form of object tracking and cognitive processing speed. However, it is unclear what specifically the MOA is measuring and, relatedly, it is unclear which other attentional mechanisms may correlate to MOA performance (e.g. working memory function, sustained attention, visuomotor control). It would also be important to explore these elements in relation to broader theories of visual attention selection. We discuss the Biased Competition Theory to offer possible links to visual attention theory – but much more rigorous testing would be needed to provide more concrete links between, for example, the BCT, but others too (such as the Attention Network as described by Fan et al., (2002)).

Whilst we demonstrate some evidence of test–retest reliability, it is important to highlight that this was a moderate correlation. There could be several reasons for this. From the more extreme data points in Fig. 5, some of this variability could be explained by a learning effect between testing points where we see some individuals perform a good deal better at the retest. However, there appears to also be a somewhat equal number who did quite a bit worse on the second testing stage. Reasons for either the increase or decrease could be, for example, differing levels of motivation (Deci et al., 1999; Van Lange et al., 2011) or effects on cognition due to aspects such as testing on a different time of day (Wesensten et al., 1990), varying levels of fatigue (Kronholm et al., 2009) or even potentially prior levels of food/liquid intake (Adan, 2012). We also propose that possible different speeds of stimuli at test and re-test due to chance could have contributed. The open-source nature of this task will allow researchers to test and validate the ball speeds for their own purposes (e.g., if using with older adults).

Beyond this, one potential line of future research to take would be that of attentional assessment. In the case of driving, there is a safety element here that should not be ignored. We found evidence that better performance in the MOA related to simulated driving performance, and, potentially, an element of road safety – hazard perception. It would be important to validate the MOA in relation to driving safety in order to begin research into developing the MOA as an assessment tool. It is important to highlight that the results here are in relation to *simulated* driving performance. Whilst driving simulations can often be used a good measure of relative validity (e.g. Underwood et al., 2011) we should be cautious in extending our results to on-road performance.

We end our discussion by imploring future research to take advantage of this open-source task and use the MOA as an attention measure in other, wider, domains and other ‘everyday’ tasks as some have already begun to do (e.g. in lifeguarding: Laxton, Mackenzie & Crundall, *submitted*).

## Conclusion

The MOA task measures attention for action and relates to real world tasks such as driving and sport. This study investigated 1) how performance in MOA predicted individual differences in simulated driving performance, 2) the relationship to attentional tracking and cognitive processing speed and 3) how MOA performance might identify individual differences in sporting expertise. MOA performance correlated with driving performance where better performance in MOA predicted better driving scores (Study 1). This study also found a moderate test–retest reliability for the MOA task. Study 2 found that those that play sports also performed better at MOA suggesting the utility of the MOA beyond driving in measuring visual attention in real world skills. We also showed that the MOA was related to cognitive processing speed and MOT performance which may demonstrate elements of the MOA’s compositional nature and offer some construct validity. We recommend that researchers consider using the MOA task to investigate attention for action in other applied domains and visual attention research.



## References

[CR1] Adan A (2012). Cognitive performance and dehydration. Journal of the American College of Nutrition.

[CR2] Aksan N, Sager L, Hacker S, Lester B, Dawson J, Rizzo M, Ebe K, Foley J (2017). Individual differences in cognitive functioning predict effectiveness of a heads-up lane departure warning for younger and older drivers. Accident Analysis & Prevention.

[CR3] Alberti, C. F., Horowitz, T., Bronstad, P. M., Bowers, A. R. (2014) Visual attention measures predict pedestrian detection in central field loss: a pilot study. *PLoS One 9*(2): e89381. 10.1371/journal.pone.0089381 10.1371/journal.pone.0089381 PMC3928437 24558495

[CR4] Ball K, Owsley C, Sloane ME, Roenker DL, Bruni JR (1993). Visual attention problems as a predictor of vehicle crashes in older drivers. Invest Ophthalmol Vis Sci.

[CR5] Ball K, Roenker DL, Bruni JR (1990). Developmental changes in attention and visual search throughout adulthood. Advances in Psychology.

[CR6] Bédard M, Campbell S, Riendeau J, Maxwell H, Weaver B (2016). Visual-cognitive tools used to determine fitness-to-drive may reflect normal aging. Clinical and Experimental Optometry.

[CR7] Bedard M, Weaver B, Dārzin P, Porter MM (2008). Predicting driving performance in older adults: we are not there yet!. Traffic Injury Prevention.

[CR8] Bowers, A R, Anastasio, J., Howe, P., O’Connor, M., Hollis, A., Kapust, L., Bronstad, M., & Horowitz, T. (2011). Dynamic attention as a predictor of driving performance in clinical populations: preliminary results. Proceedings of the Sixth International Driving Symposium on Human Factors in Driver Assessment, Training and Vehicle Design, June 27-30, 2011, Olympic Valley — Lake Tahoe, California. Iowa City, IA: Public Policy Center, University of Iowa, 2011:307–313. 10.17077/drivingassessment.1413

[CR9] Bowers AR, Anastasio RJ, Sheldon SS, O’Connor MG, Hollis AM, Howe PD, Horowitz TS (2013). Can we improve clinical prediction of at-risk older drivers?. Accident Analysis & Prevention.

[CR10] Cavanagh P, Alvarez GA (2005). Tracking multiple targets with multifocal attention. Trends in Cognitive Sciences.

[CR11] Clay OJ, Wadley VG, Edwards JD, ROTH DL, Roenker DL, Ball KK (2005). Cumulative meta-analysis of the relationship between useful field of view and driving performance in older adults: Current and future implications. Optometry & Vision Science.

[CR12] Cohen, J. (1988). *Statistical power analysis for the behavioural sciences. Hillsdale, NJ: Laurence Erlbaum Associates*. Inc, Publishers

[CR13] Crundall D (2009). The deceleration detection flicker test: A measure of experience?. Ergonomics.

[CR14] Crundall D (2016). Hazard prediction discriminates between novice and experienced drivers. Accident Analysis & Prevention.

[CR15] Crundall D, Chapman P, Trawley S, Collins L, van Loon E, Andrews B, Underwood G (2012). Some hazards are more attractive than others: drivers of varying experience respond differently to different types of hazard. Accid Anal Prev.

[CR16] Crundall D, Underwood G (1998). Effects of experience and processing demands on visual information acquisition in drivers. Ergonomics.

[CR17] Deci EL, Koestner R, Ryan RM (1999). A meta-analytic review of experiments examining the effects of extrinsic rewards on intrinsic motivation. Psychological Bulletin.

[CR18] Desimone R, Duncan J (1995). Neural mechanisms of selective visual attention. Annual Review of Neuroscience.

[CR19] Dingus, T. A., Klauer, S., Neale, V., Petersen, A., Lee, S., Sudweeks, J., . . . Gupta, S. (2006). The 100-car naturalistic driving study, Phase II-results of the 100-car field experiment (National Highway and Traffic Safety Administration Report No. DOT HS 810 593). Washington, DC: National Highway and Traffic Safety Administration.

[CR20] Downing PE (2000). Interactions between visual working memory and selective attention. Psychological Science.

[CR21] Eccles DW (2006). Thinking outside of the box: The role of environmental adaptation in the acquisition of skilled and expert performance. Journal of Sports Sciences.

[CR22] Engle RW (2002). Working memory capacity as executive attention. Current Directions in Psychological Science.

[CR23] Ericsson KA, Krampe RT, Tesch-Römer C (1993). The role of deliberate practice in the acquisition of expert performance. Psychological Review.

[CR24] Fan J, Gu X, Guise KG, Liu X, Fossella J, Wang H, Posner MI (2009). Testing the behavioral interaction and integration of attentional networks. Brain and Cognition.

[CR25] Fan J, McCandliss BD, Sommer T, Raz A, Posner MI (2002). Testing the efficiency and independence of attentional networks. Journal of Cognitive Neuroscience.

[CR26] Fehd HM, Seiffert AE (2008). Eye movements during multiple object tracking: Where do participants look?. Cognition.

[CR27] Foulsham T, Walker E, Kingstone A (2011). The where, what and when of gaze allocation in the lab and the natural environment. Vision Research.

[CR28] Fry AF, Hale S (2000). Relationships among processing speed, working memory, and fluid intelligence in children. Biological Psychology.

[CR29] Furley PA, Memmert D (2012). Working memory capacity as controlled attention in tactical decision making. Journal of Sport and Exercise Psychology.

[CR30] Furley, P., & Memmert, D. (2013). “Whom should I pass to?” the more options the more attentional guidance from working memory. *Plos One*, *8*(5), e62278. 10.1371/journal.pone.0062278 10.1371/journal.pone.0062278 PMC3642109 23658719

[CR31] Furley P, Memmert D, Heller C (2010). The dark side of visual awareness in sport: Inattentional blindness in a real-world basketball task. Attention, Perception, & Psychophysics.

[CR32] Furley P, Wood G (2016). Working memory, attentional control, and expertise in sports: A review of current literature and directions for future research. Journal of Applied Research in Memory and Cognition.

[CR33] Guest D, Howard CJ, Brown LA, Gleeson H (2015). Aging and the rate of visual information processing. Journal of Vision.

[CR34] Guest, D., Mackenzie, A., Howard, C. J., Badham, S., & Brown, L. (2017). Number of objects and number of features influence the extent of age related differences in visual information processing. Abstract from Experimental Psychology Society, Reading, United Kingdom

[CR35] Hambrick, D. Z., Macnamara, B. N., Campitelli, G., Ullén, F., & Mosing, M. A. (2016). Beyond born versus made: A new look at expertise. In Psychology of learning and motivation (Vol. 64, pp. 1–55). Academic Press.

[CR36] Harris, D. J., Wilson, M. R., Crowe, E. M., & Vine, S. J. (2020). Examining the roles of working memory and visual attention in multiple object tracking expertise. *Cognitive Processing, 21*(2), 209–222. 10.1007/s10339-020-00954-y PMC7203592 32016685

[CR37] Hayhoe M, Ballard D (2005). Eye movements in natural behavior. Trends in Cognitive Sciences.

[CR38] Hommel B (2010). Grounding attention in action control: The intentional control of selection.

[CR39] Howard CJ, Holcombe AO (2008). Tracking the changing features of multiple objects: Progressively poorer perceptual precision and progressively greater perceptual lag. Vision Research.

[CR40] Howard CJ, Rollings V, Hardie A (2017). Sustained attention to objects’ motion sharpens position representations: Attention to changing position and attention to motion are distinct. Vision Research.

[CR41] Howard, C. J., Uttley, J., & Andrews, S. (2018). Team ball sport participation is associated with performance in two sustained visual attention tasks: Position monitoring and target identification in rapid serial visual presentation streams. *Progress in Brain Research, 240*, 53–69. 10.1016/bs.pbr.2018.09.001 30390841

[CR42] Huang L, Pashler H (2007). Working memory and the guidance of visual attention: Consonance-driven orienting. Psychonomic Bulletin & Review.

[CR43] Humphreys GW, Yoon EY, Kumar S, Lestou V, Kitadono K, Roberts KL, Riddoch MJ (2010). Attention and its coupling to action. British Journal of Psychology.

[CR44] Jackson L, Chapman P, Crundall D (2009). What happens next? Predicting other road users’ behaviour as a function of driving experience and processing time. Ergonomics.

[CR45] Karimi M, Hedner J, Zou D, Eskandari D, Lundquist A-C, Grote L (2015). Attention deficits detected in cognitive tests differentiate between sleep apnea patients with or without a motor vehicle accident. Sleep Medicine.

[CR46] Konstantopoulos P, Chapman P, Crundall D (2010). Driver’s visual attention as a function of driving experience and visibility. Using a driving simulator to explore drivers’ eye movements in day, night and rain driving. Accid Anal Prev.

[CR47] Konstantopoulos P, Chapman P, Crundall D (2012). Exploring the ability to identify visual search differences when observing drivers’ eye movements. Transportation Research Part F: Traffic Psychology and Behaviour.

[CR48] Kountouriotis GK, Floyd RC, Gardner PH, Merat N, Wilkie RM (2012). The role of gaze and road edge information during high-speed locomotion. Journal of Experimental Psychology: Human Perception and Performance.

[CR49] Kronholm E, Sallinen M, Suutama T, Sulkava R, Era P, Partonen T (2009). Self-reported sleep duration and cognitive functioning in the general population. Journal of Sleep Research.

[CR50] Land MF (2006). Eye movements and the control of actions in everyday life. Prog Retin Eye Res.

[CR51] Land MF, Hayhoe M (2001). In what ways do eye movements contribute to everyday activities?. Vision Research.

[CR52] Land, M. F., & Lee, D. N. (1994). Where we look when we steer. *Nature, 369*(6483), 742–744. 10.1038/369742a0 8008066

[CR53] Land MF, McLeod P (2000). From eye movements to actions: how batsmen hit the ball. Nature Neuroscience.

[CR54] Lee JD (2008). Fifty years of driving safety research. Human Factors: The Journal of the Human Factors and Ergonomics Society.

[CR55] Lee YM, Miller K, Crundall D, Sheppard E (2020). Cross-cultural effects on detecting multiple sources of driving hazard: Evidence from the deceleration detection flicker test. Transportation Research Part F: Traffic Psychology and Behaviour.

[CR56] Lehtonen E, Lappi O, Koirikivi I, Summala H (2014). Effect of driving experience on anticipatory look-ahead fixations in real curve driving. Accid Anal Prev.

[CR57] Liebherr M, Antons S, Schweig S, Maas N, Schramm D, Brand M (2019). Driving performance and specific attentional domains. Transportation Research Interdisciplinary Perspectives.

[CR58] Louie JF, Mouloua M (2019). Predicting distracted driving: the role of individual differences in working memory. Applied Ergonomics.

[CR59] Mackenzie, A. K., & Harris, J. M. (2015). Eye movements and hazard perception in active and passive driving. *Visual Cognition, 23*(6), 736–757. 10.1080/13506285.2015.1079583 PMC4673545 26681913

[CR60] Mackenzie AK, Harris JM (2017). A link between attentional function, effective eye movements, and driving ability. Journal of Experimental Psychology: Human Perception and Performance.

[CR61] Meng, F. W., Yao, Z. F., Chang, E. C., & Chen, Y. L. (2019). Team sport expertise shows superior stimulus-driven visual attention and motor inhibition. *PloS One, 14*(5), e0217056. 10.1371/journal.pone.0217056 PMC6519903 31091297

[CR62] Michaels J, Chaumillon R, Nguyen-Tri D, Watanabe D, Hirsch P, Bellavance F, Giraudet G, Bernardin D, Faubert J (2017). Driving simulator scenarios and measures to faithfully evaluate risky driving behavior: a comparative study of different driver age groups. PLoS One.

[CR64] Oksama L, Hyönä J (2016). Position tracking and identity tracking are separate systems: Evidence from eye movements. Cognition.

[CR65] Oreyzi HR, Haghayegh SA (2010). Psychometric properties of the Manchester driving behavior questionnaire. Payesh (Health Monitor).

[CR66] Overney, L. S., Blanke, O., & Herzog, M. H. (2008). Enhanced temporal but not attentional processing in expert tennis players. *PLoS One, 3*(6), e2380. 10.1371/journal.pone.0002380 PMC2398771 18545661

[CR67] Owsley C, McGwin G (2010). Vision and driving. Vision Res.

[CR68] Owsley C (2013). Visual processing speed. Vision Research.

[CR69] Pelz J, Hayhoe M, Loeber R (2001). The coordination of eye, head, and hand movements in a natural task. Experimental Brain Research.

[CR70] Peng P, Miller AC (2016). Does attention training work? A selective meta-analysis to explore the effects of attention training and moderators. Learning and Individual Differences.

[CR71] Pope CN, Ross LA, Stavrinos D (2016). Association between executive function and problematic adolescent driving. Journal of Developmental and Behavioral Pediatrics: JDBP.

[CR72] Posner MI, Rothbart MK, Tang Y-Y (2015). Enhancing attention through training. Current Opinion in Behavioral Sciences.

[CR73] Pylyshyn ZW, Storm RW (1988). Tracking multiple independent targets: Evidence for a parallel tracking mechanism. Spatial Vision.

[CR74] Qiu F, Pi Y, Liu K, Li X, Zhang J, Wu Y (2018). Influence of sports expertise level on attention in multiple object tracking. PeerJ.

[CR75] Reason J, Manstead A, Stradling S, Baxter J, Campbell K (1990). Errors and violations on the roads: a real distinction?. Ergonomics.

[CR76] Risko, E. F., Laidlaw, K. E., Freeth, M., Foulsham, T., & Kingstone, A. (2012). Social attention with real versus reel stimuli: toward an empirical approach to concerns about ecological validity. *Frontiers in Human Nneuroscience, 6*, 143. 10.3389/fnhum.2012.00143 PMC3360477 22654747

[CR77] Roca J, Crundall D, Moreno-Rios S, Castro C, Lupianez J (2013). The influence of differences in the functioning of the neurocognitive attentional networks on drivers’ performance. Accid Anal Prev.

[CR78] Roca J, Tejero P, Insa B (2018). Accident ahead? Difficulties of drivers with and without reading impairment recognising words and pictograms in variable message signs. Applied Ergonomics.

[CR79] Ross LA, Edwards JD, O’Connor ML, Ball KK, Wadley VG, Vance DE (2016). The transfer of cognitive speed of processing training to older adults’ driving mobility across 5 years. Journals of Gerontology Series B: Psychological Sciences and Social Sciences.

[CR80] Salthouse TA (1992). Influence of processing speed on adult age differences in working memory. Acta Psychologica.

[CR81] Salthouse TA (2005). Relations between cognitive abilities and measures of executive functioning. Neuropsychology.

[CR82] Scharfen, H. E., & Memmert, D. (2019). The relationship between cognitive functions and sport-specific motor skills in elite youth soccer players. *Frontiers in Psychology, 10*, 817. 10.3389/fpsyg.2019.00817 PMC6494938 31105611

[CR83] Shahar A, Alberti CF, Clarke D, Crundall D (2010). Hazard perception as a function of target location and the field of view. Accid Anal Prev.

[CR84] Tabibi Z, Borzabadi HH, Stavrinos D, Mashhadi A (2015). Predicting aberrant driving behaviour: The role of executive function. Transportation Research Part F: Traffic Psychology and Behaviour.

[CR85] Tatler BW, Hayhoe MM, Land MF, Ballard DH (2011). Eye guidance in natural vision: reinterpreting salience. Journal of Vis.

[CR86] Tejero P, Insa B, Roca J (2019). Reading Traffic Signs While Driving: Are Linguistic Word Properties Relevant in a Complex, Dynamic Environment?. Journal of Applied Research in Memory and Cognition.

[CR87] Thornton IM, Bülthoff HH, Horowitz TS, Rynning A, Lee S-W (2014). Interactive multiple object tracking (iMOT). PloS One.

[CR88] Thornton IM, Horowitz TS (2015). Does action disrupt multiple object tracking (MOT)?. Psihologija.

[CR89] Underwood G, Crundall D, Chapman P (2002). Selective searching while driving: the role of experience in hazard detection and general surveillance. Ergonomics.

[CR90] Underwood G, Crundall D, Chapman P (2011). Driving simulator validation with hazard perception. Transportation Research Part F: Traffic Psychology and Behaviour.

[CR91] Underwood G, Phelps N, Wright C, Van Loon E, Galpin A (2005). Eye fixation scanpaths of younger and older drivers in a hazard perception task. Ophthalmic and Physiological Optics.

[CR92] Van Lange PAM, Schippers M, Balliet D (2011). Who volunteers in psychology experiments? An empirical review of prosocial motivation in volunteering. Personality and Individual Differences.

[CR93] Velichkovsky BM, Rothert A, Kopf M, Dornhöfer SM, Joos M (2002). Towards an express-diagnostics for level of processing and hazard perception. Transportation Research Part F: Traffic Psychology and Behaviour.

[CR94] Voss MW, Kramer AF, Basak C, Prakash RS, Roberts B (2010). Are expert athletes ‘expert’in the cognitive laboratory? A meta-analytic review of cognition and sport expertise. Applied Cognitive Psychology.

[CR95] Weaver B, Bédard M, McAuliffe J, Parkkari M (2009). Using the Attention Network Test to predict driving test scores. Accident Analysis & Prevention.

[CR96] Wechsler, D., & De Lemos, M. M. (1981). Wechsler adult intelligence scale-revised.

[CR97] Wesensten NJ, Badia P, Harsh J (1990). Time of day, repeated testing, and interblock interval effects on P300 amplitude. Physiology & Behavior.

[CR98] Wood G, Hartley G, Furley PA, Wilson MR (2016). Working memory capacity, visual attention and hazard perception in driving. Journal of Applied Research in Memory and Cognition.

[CR99] Zelinsky GJ, Neider MB (2008). An eye movement analysis of multiple object tracking in a realistic environment. Visual Cognition.

[CR100] Zhao N, Mehler B, Reimer B, D’Ambrosio LA, Mehler A, Coughlin JF (2012). An investigation of the relationship between the driving behavior questionnaire and objective measures of highway driving behavior. Transportation Research Part F: Traffic Psychology and Behaviour.

